# Fungal genome and mating system transitions facilitated by chromosomal translocations involving intercentromeric recombination

**DOI:** 10.1371/journal.pbio.2002527

**Published:** 2017-08-11

**Authors:** Sheng Sun, Vikas Yadav, R. Blake Billmyre, Christina A. Cuomo, Minou Nowrousian, Liuyang Wang, Jean-Luc Souciet, Teun Boekhout, Betina Porcel, Patrick Wincker, Joshua A. Granek, Kaustuv Sanyal, Joseph Heitman

**Affiliations:** 1 Department of Molecular Genetics and Microbiology, Duke University Medical Center, Durham, North Carolina, United States of America; 2 Molecular Biology and Genetics Unit, Jawaharlal Nehru Centre for Advanced Scientific Research, Bangalore, India; 3 Broad Institute of MIT and Harvard, Cambridge, Massachusetts, United States of America; 4 Lehrstuhl für Allgemeine und Molekulare Botanik, Ruhr-Universität Bochum, Bochum, Germany; 5 Université de Strasbourg, CNRS UMR7156, Strasbourg, France; 6 Westerdijk Fungal Biodiversity Institute, Utrecht, The Netherlands; 7 Institute for Biodiversity and Ecosystem Dynamics (IBED), University of Amsterdam, Amsterdam, The Netherlands; 8 Commissariat à l'Energie Atomique (CEA), Institut de Génomique (IG), Genoscope, Evry, France; 9 Université d'Evry, UMR 8030, Evry, France; 10 Centre National de Recherche Scientifique (CNRS), UMR 8030, Evry, France; University College Dublin, Ireland

## Abstract

Species within the human pathogenic *Cryptococcus* species complex are major threats to public health, causing approximately 1 million annual infections globally. *Cryptococcus amylolentus* is the most closely known related species of the pathogenic *Cryptococcus* species complex, and it is non-pathogenic. Additionally, while pathogenic *Cryptococcus* species have bipolar mating systems with a single large mating type (*MAT*) locus that represents a derived state in Basidiomycetes, *C*. *amylolentus* has a tetrapolar mating system with 2 *MAT* loci (*P/R* and *HD*) located on different chromosomes. Thus, studying *C*. *amylolentus* will shed light on the transition from tetrapolar to bipolar mating systems in the pathogenic *Cryptococcus* species, as well as its possible link with the origin and evolution of pathogenesis. In this study, we sequenced, assembled, and annotated the genomes of 2 *C*. *amylolentus* isolates, CBS6039 and CBS6273, which are sexual and interfertile. Genome comparison between the 2 *C*. *amylolentus* isolates identified the boundaries and the complete gene contents of the *P/R* and *HD MAT* loci. Bioinformatic and chromatin immunoprecipitation sequencing (ChIP-seq) analyses revealed that, similar to those of the pathogenic *Cryptococcus* species, *C*. *amylolentus* has regional centromeres (*CEN*s) that are enriched with species-specific transposable and repetitive DNA elements. Additionally, we found that while neither the *P/R* nor the *HD* locus is physically closely linked to its centromere in *C*. *amylolentus*, and the regions between the *MAT* loci and their respective centromeres show overall synteny between the 2 genomes, both *MAT* loci exhibit genetic linkage to their respective centromere during meiosis, suggesting the presence of recombinational suppressors and/or epistatic gene interactions in the *MAT*-*CEN* intervening regions. Furthermore, genomic comparisons between *C*. *amylolentus* and related pathogenic *Cryptococcus* species provide evidence that multiple chromosomal rearrangements mediated by intercentromeric recombination have occurred during descent of the 2 lineages from their common ancestor. Taken together, our findings support a model in which the evolution of the bipolar mating system was initiated by an ectopic recombination event mediated by similar repetitive centromeric DNA elements shared between chromosomes. This translocation brought the *P/R* and *HD* loci onto the same chromosome, and further chromosomal rearrangements then resulted in the 2 *MAT* loci becoming physically linked and eventually fusing to form the single contiguous *MAT* locus that is now extant in the pathogenic *Cryptococcus* species.

## Author summary

This manuscript explores the evolution of the genomic regions encoding the mating type loci of basidiomycetous fungi. Typically, the mating system is tetrapolar, meaning that it is composed of 2 unlinked mating type (*MAT*) loci (*P/R* and *HD*) that are located on different chromosomes. However, species with bipolar mating systems, in which the *P/R* and *HD* loci are located on the same chromosome, have also been identified. Tetrapolar and bipolar species are often closely related, suggesting the transition between these 2 mating systems might occur frequently. For example, the species within the human fungal pathogenic *Cryptococcus* species complex have bipolar mating systems, with 1 large *MAT* locus that appears to be a fusion product of the *P/R* and *HD* loci. On the other hand, the species that is the closest outgroup to these pathogenic species, *Cryptococcus amylolentus*, appears to have a classic tetrapolar mating system. Interestingly, the 2 *MAT* loci of *C*. *amylolentus* exhibit centromeric linkage during meiosis, and as a consequence, their resulting meiotic segregation pattern differs from other regions of the genome. Additionally, both pathogenic and non-pathogenic species are found to have large regional centromeres enriched with transposable and repetitive elements. Our genome comparison analyses indicated that these regional centromeres underwent ectopic recombination during the evolution of these 2 lineages. Based on these observations, we propose a model for the transition from the tetrapolar mating system in non-pathogenic *C*. *amylolentus* to the bipolar mating system in its related pathogenic species that is initiated by intercentromeric ectopic recombination, followed by chromosomal rearrangements. These events moved the 2 *MAT* loci closer to each other and eventually fused them to form a single *MAT* locus. This model is also consistent with recent findings on the organization of *MAT* loci in other basidiomycetous species.

## Introduction

In the Basidiomycota, mating type is typically determined by a tetrapolar mating system that comprises 2 mating type (*MAT*) loci, one encoding pheromones and pheromone receptor genes (the *P/R* locus) and the other encoding transcription factors that govern sexual development (the *HD* locus). In most cases, these 2 *MAT* loci are located on different chromosomes and segregate independently during sexual reproduction [1–3]. However, in some basidiomycetous species the 2 *MAT* loci became physically linked and are now located on the same chromosome. For example, the smut fungus *Ustilago maydis* has a tetrapolar mating system where the *P/R* and *HD MAT* loci are located on different chromosomes, whereas its sister species *Ustilago hordei* and *Ustilago bromivora* have bipolar mating systems in which the *P/R* and *HD* loci are located on the same chromosome and linked, and the distances separating the 2 *MAT* loci are approximately 500 kb and 180 kb, respectively, in the 2 species [4,5]. The *P/R* and *HD* loci are also linked in several species of human skin fungal pathogens belonging to the *Malassezia* species complex, including *M*. *sympodialis*, *M*. *globosa*, *M*. *furfur*, and *M*. *yamatoensis*, with distances between the 2 *MAT* loci ranging from approximately 140 kb to 580 kb in different species [6,7]. Additionally, *M*. *sympodialis* represents an intermediate state between tetrapolar and bipolar, and in this case the *P/R* and *HD* loci are linked but still undergo recombination. The close relationship between species with classic tetrapolar mating system and both the bipolar and “pseudo-bipolar” mating system where the *P/R* and *HD* loci are physically linked suggests that transitions between the 2 mating systems occurred frequently during evolution, although the exact underlying mechanisms responsible for this type of chromosomal rearrangements are not fully understood.

It is known that active transposable elements, as well as repetitive sequences, can mediate chromosomal rearrangements, such as inversions and translocations through ectopic recombination between non-allelic homologous sequences, which results in genome instability [8–11]. As a result, organisms have evolved a variety of defense mechanisms to keep the activities of transposable elements and their detrimental consequences under control [12,13]. While transposons can be present in any part of the genome, the centromere is one chromosomal region that is typically enriched with transposable and repetitive elements. The heterochromatic nature of centromeres may suppress expression of genes required for transposition, and thus transposons may become trapped within the centromere. There is a broad range of complexity in the length, composition of DNA sequence, and organization of the sequence elements of the centromere that varies from simple genetically defined “point” centromeres found in the budding yeast *Saccharomyces cerevisiae* to complex epigenetically determined regional centromeres in most other organisms. The length of a regional centromere ranges from a few kilobases (e.g., *Candida albicans*) to tens of kilobases (e.g., *Cryptococcus neoformans* and *Schizosaccharomyces pombe*), and even up to hundreds of kilobases in plants and animals [14]. It has long been thought that centromeres are highly repressed for recombination. However, several recent studies suggest this is not always the case, and that recombination within centromeres can occur at frequencies higher than previously appreciated [15–18]. Additionally, studies of the human fungal pathogen *Candida tropicalis*, as well as those in the pathogenic *Cryptococcus* species complex, provide evidence that chromosomal arm exchanges have resulted from intercentromeric ectopic recombination during the evolution of these species. These recombination events were mediated by the highly similar transposable/repetitive elements present in regional centromeres (*CEN*s) of different chromosomes of a given species [19,20]. Thus, similar intercentromeric ectopic recombination might drive transitions between tetrapolar mating systems and pseudo-bipolar mating systems in basidiomycetous species by bringing together the 2 chromosomal arms bearing the *P/R* and *HD* loci.

Species within the human basidiomycetous fungal pathogen *Cryptococcus* species complex are major threats to public health, causing about 1 million infections and more than 600,000 deaths globally each year [21]. The major infectious propagules of *Cryptococcus* species are desiccated yeast cells and basidiospores, which are produced during sexual reproduction. Specifically, the zygotes formed between compatible mating partners grow as hyphae, and eventually the end of the hypha expands and forms a basidium, within which nuclear fusion and meiosis occur, producing 4 meiotic products. These 4 meiotic products then undergo repeated rounds of mitosis and generate 4 chains of basidiospores on the surface of the basidium. One unique feature of the human pathogenic *Cryptococcus* species is that all have a bipolar mating system defined by a single *MAT* locus carrying both the *P/R* and the *HD* genes. While it has been hypothesized that the *MAT* locus in the pathogenic *Cryptococcus* species resulted from the fusion of the ancestral *P/R* and *HD* loci through ectopic recombination, it remains unclear how this transition occurred. *Cryptococcus amylolentus* is the species most closely related to the pathogenic *Cryptococcus* species complex, and it is non-pathogenic. Additionally, *C*. *amylolentus* has a tetrapolar mating system with 2 *MAT* loci (*P/R* and *HD*) located on different chromosomes [22], in contrast to the bipolar mating system of the pathogenic *Cryptococcus* species. Previous studies demonstrated that the genes located within the *MAT* locus of the pathogenic *Cryptococcus* species are also located in the close vicinity of the key *MAT* defining genes (such as the pheromone and pheromone receptor genes of the *P/R* locus and the homeodomain transcription factors of the *HD* locus) in *C*. *amylolentus* [22]. Because the boundaries of the *MAT* loci have not been definitively established, it is not clear whether these genes are also located within the *MAT* loci in *C*. *amylolentus*. Thus, we determined the complete *C*. *amylolentus* genomes, including the characterization of the *MAT* loci (*P/R* and *HD*) and their segregation patterns during meiosis, as well as mapping and precise assembly of the centromeres, to unravel the sequence of events that led to genomic evolution of these closely related species and the transition between the tetrapolar and bipolar mating systems. We found that the 2 *MAT* loci in *C*. *amylolentus* are genetically linked to their respective *CEN*s, which are composed of species-specific transposable elements and their remnants. Additionally, comparing the genomes of *C*. *amylolentus* and its closely related pathogenic *Cryptococcus* species, we found evidence of chromosomal translocations mediated by the centromeres, including the chromosomes on which the *MAT* loci reside. We propose a model in which the transition between tetrapolar and bipolar mating systems was initiated by intercentromeric recombination mediated by highly similar transposable/repetitive centromeric DNA elements shared between chromosomes, resulting in chromosomal translocations that established the initial linkage between the *P/R* and *HD* loci. We also discuss our findings in the context of chromosomal rearrangement and the evolution of transposon-rich regional centromeres.

## Results

### Genome assemblies and the *MAT* loci of *Cryptococcus amylolentus*

**Fig 1:**
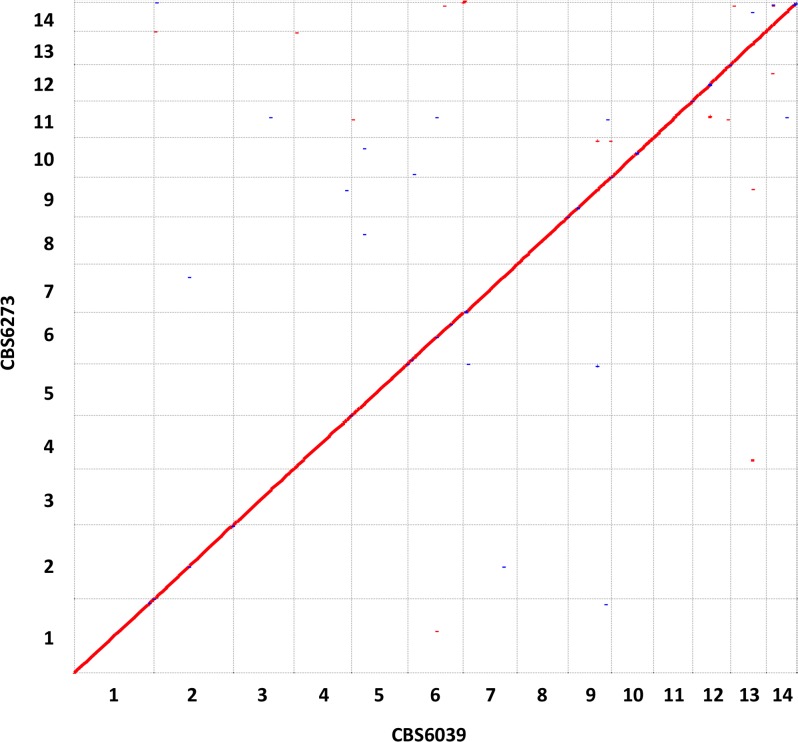
Genome comparison between the 2 *Cryptococcus amylolentus* isolates CBS6039 and CBS6273. Shown here are results of dot plot visualization of alignments between the 2 *C*. *amylolentus* genomes with Nucmer maximum gap size set at 10. Data used to generate the figure can be found at NCBI BioProject with accession no. PRJNA200571 and EBI with study accession no. PRJEB19939.

We sequenced the genomes of the 2 *C*. *amylolentus* isolates, CBS6039 and CBS6273, which are interfertile and produce viable basidiospores, using Roche/454 (only for CBS6039), Illumina, and PacBio platforms. Hybrid assembly of this data resulted in 2 genomes, each approximately 20.3 Mb in size and containing 14 chromosomes, with telomeric tandem arrays of C_(4,6)_GCTAA identified at the ends of 13 chromosomes in CBS6039 and 11 chromosomes in CBS6273, respectively (Table 1). This is consistent with the numbers and sizes of the chromosomes in these 2 strains as visualized by contour-clamped homogeneous electric field (CHEF) electrophoresis. Chromoblot analysis using probes targeting the opposite ends of each chromosome confirmed the chromosomal organization in the assemblies (S1 Fig). For chromosomes 10 and 11, on which the *P/R* and *HD* loci are located, respectively, our genetic analyses showed that markers spanning each chromosome form a single linkage group during meiosis, and thus, confirming the assemblies of these 2 chromosomes. With the exception of the *MAT* loci, the genomes of strains CBS6039 and CBS6273 are in overall synteny, with only small-scale insertions/deletions and chromosomal rearrangements (e.g., translocations and inversions) identified between the 2 genomes (Fig 1 and S1 Table). Specifically, of the 245 insertion/deletion and inversion events identified between the CBS6039 and CBS6273 genomes, 77 are larger than 500 bp and 51 are larger than 1 kb in size. Compared to the genome of CBS6273, the genome of CBS6039 has only 3 insertions and 1 deletion that are larger than 10 kb in size, and they are located on chromosomes 2, 4, 5, and 11, respectively. Interestingly, the >10 kb deletion on CBS6039 chromosome 11 is located in a region that flanks the centromere and lies between the *HD MAT* locus and the centromere (S1 Table).

**Table 1:** Summary of the genome assemblies of *Cryptococcus amylolentus* strains CBS6039 and CBS6273.

CBS6039		CBS6273	
Chromosome	Size (bp)	Supercontig	Size (bp)
1	2,227,464	2	2,226,283
2	2,220,979	1	2,225,154
3	1,707,710	3	1,710,600
4	1,608,529	4	1,608,917
5	1,576,773	5	1,560,917
6	1,545,655	6	1,529,399
7	1,509,211	7	1,455,708
8	1,429,001	8	1,428,089
9	1,219,259	9	1,210,710
10	1,165,603	10	1,178,207
11	1,107,941	11	1,127,347
12	1,061,032	12	1,092,861
13	1,002,165	13	1,005,782
14	832,781	14	854,032
Total	20,254,996	Total	20,294,622

Annotation of the CBS6039 genome, with the assistance of the RNA sequencing (RNA-seq) data, identified 8,248 protein models. Compared to the species within the human pathogenic *Cryptococcus* species complex, genes of 4 metabolic pathways show significant expansion in the *C*. *amylolentus* genomes (S2 Table). Additionally, the 2 *C*. *amylolentus* isolates also differ in the number of genes in these enriched pathways. For example, there are 56 and 30 components in the NAD_binding_10 and NmrA-like_family pathways, respectively, in the genome of strain CBS6039, while there are 54 and 29 components in these 2 pathways, respectively, in the genome of strain CBS6273 (S2 Table).

**Fig 2:**
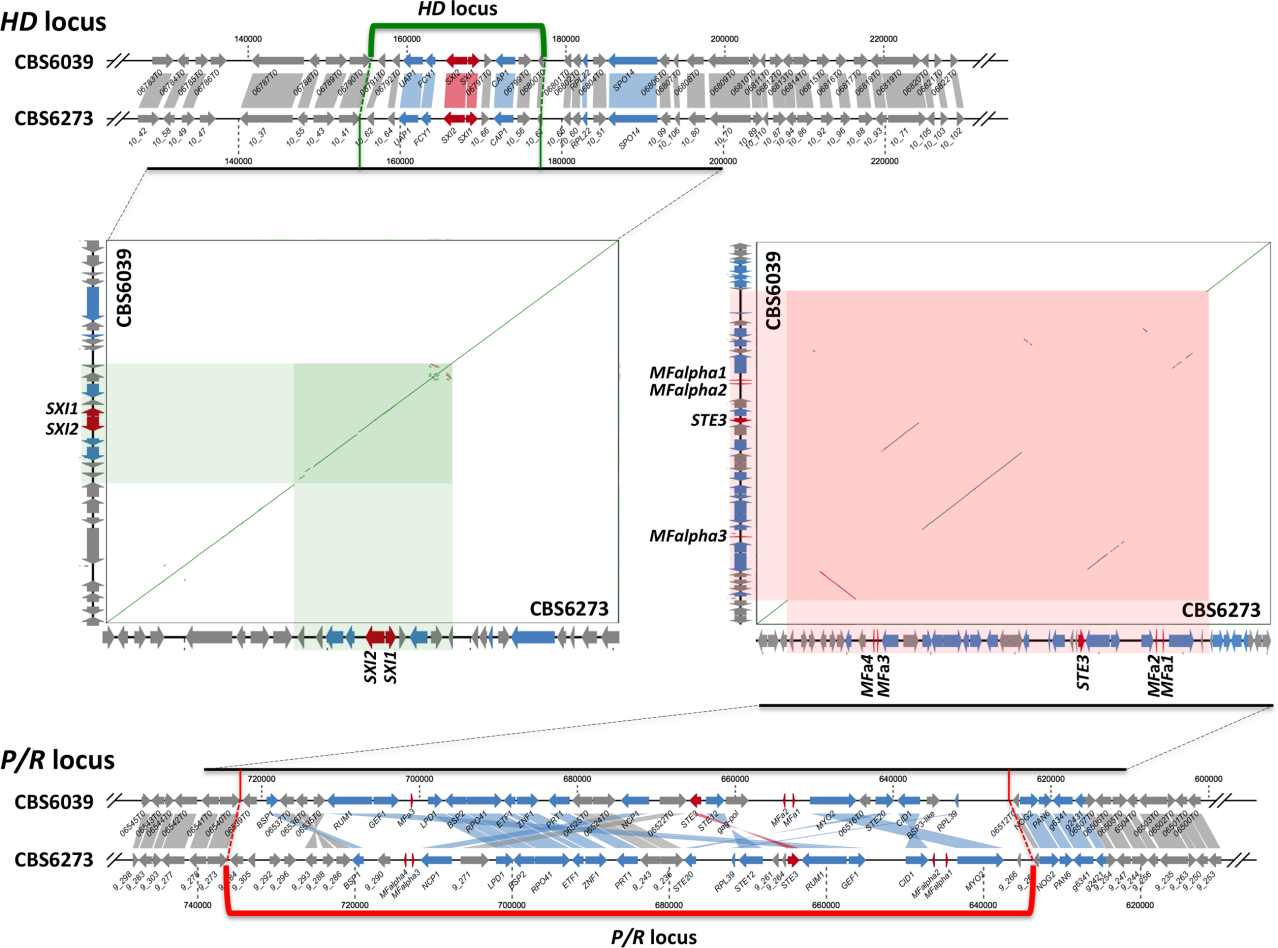
Synteny map of the *MAT* loci in *Cryptococcus amylolentus* and closely related species. Shown at the top and the bottom are results of synteny analyses between sequences from strains CBS6039 and CBS6273 for the *HD* and *P/R* loci, respectively. Red color highlights the genes that define the *HD* locus (*SXI1* and *SXI2*) and *P/R* locus (mating pheromones and *STE3*); blue and gray colors highlight the genes that are present or absent from the mating type (*MAT*) locus in the human pathogenic *Cryptococcus* species complex, respectively. Shown in the middle are results of dot plot analyses between strains CBS6039 and CBS6273 for the chromosomal regions encompassing the genes that define the *HD* (*SXI1* and *SXI2*) and *P/R* (mating pheromone and *STE3*) loci, respectively. Compared to the flanking regions showing complete synteny between strains CBS6039 and CBS6273, the regions highlighted in green (CBS6039 chromosome 11:155396–177087) and red (CBS6039 chromosome 10:624796–720709) exhibit significantly elevated levels of sequence divergence and chromosomal rearrangements, and define the *HD* and *P/R* loci, respectively, in *C*. *amylolentus*.

It has been previously shown that the *MAT* loci (*P/R* and *HD*) in *C*. *amylolentus* are located on different chromosomes, and the 2 isolates, CBS6039 and CBS6273, are mating-compatible, and thus have divergent alleles at the *MAT* loci [22]. However, the precise boundaries of the *MAT* loci were not known. By comparing the sequences between CBS6039 and CBS6273, we identified 1 region on each chromosome that encompasses the genes that define the *P/R* (the *MF* pheromone genes and the *STE3* pheromone receptor gene) and *HD* (the *SXI1* and *SXI2* genes) loci, respectively, and these exhibited elevated levels of sequence divergence and chromosomal rearrangements between strains CBS6039 and CBS6273. We define these regions as the *P/R* and *HD MAT* loci in *C*. *amylolentus* that are approximately 96 kb and 22 kb in size, respectively (Fig 2). It should be pointed out that the boundaries of the *HD* locus are less well-defined than those of the *P/R* locus due to the higher level of synteny and lower sequence divergence across the *HD* locus compared to the *P/R* locus. It could be that the *HD* locus only includes the *SXI1* and *SXI2* genes as in some tetrapolar species, in which case the *HD* locus will be approximately 5 kb in size. Alternatively, as shown in the dot plot in Fig 2, the *HD* locus could span not only the *SXI1* and *SXI2* genes but also 4 flanking genes on the left and 4 flanking genes on the right given sequence divergence that spans approximately 22 kb. To more precisely define the borders of the *HD MAT* locus will require finer meiotic mapping or functional studies by transformation with segments spanning the predicted *HD* locus. Assigning the flanking systemic chromosomal regions as boundaries, the *P/R* and *HD* loci in CBS6039 are estimated to be 95,914 bp and possibly as large as 21,692 bp in size, respectively (Fig 2). The combined size of the *P/R* and *HD* loci in *C*. *amylolentus* is thus similar to the *MAT* locus (approximately 120 kb) of species within the pathogenic *Cryptococcus* species complex. Additionally, of the genes that are present in the *MAT* locus of the pathogenic *Cryptococcus* species, most were found to be located within or in the vicinity of the *MAT* loci in *C*. *amylolentus* (Fig 2 and S2 Fig). It should be noted that 2 genes, *RPL22* and *SPO14*, that are located within the *MAT* locus of pathogenic *Cryptococcus* species are located in the flanking region of the *HD* locus in *C*. *amylolentus*. Additionally, the homolog of the *C*. *neoformans MAT* gene *STE11* is located on a different chromosome (chromosome 5) in the *C*. *amylolentus* genome. Conversely, there are also genes that are located within the *C*. *amylolentus MAT* loci but are missing from the *C*. *neoformans MAT* locus (Fig 2 and S2 Fig). Specifically, in strain CBS6039 there are 7 genes from the *P/R* locus and 5 genes from the *HD* locus that are located outside of the *MAT* locus in *C*. *neoformans* strain H99 genome, respectively, representing divergence that has accumulated between the 2 species after they split from their last shared common ancestor.

Taken together, we obtained high-quality genome assemblies for both *C*. *amylolentus* strains CBS6039 and CBS6273 and determined the boundaries for both the *P/R* and *HD MAT* loci, which provided a solid genomic foundation for the following analyses.

### *C*. *amylolentus* has regional centromeres that contain unique centromere-specific retrotransposons

We employed 3 independent lines of investigation and combined the results obtained to identify and characterize the centromeres of *C*. *amylolentus*: chromatin immunoprecipitation using antibodies against conserved kinetochore proteins (centromere binding protein Cse4 [CENP-A]) followed by chromatin immunoprecipitation sequencing (ChIP-seq), bioinformatics analysis to identify the longest ORF-free transposon-rich regions on each chromosome, and RNA-seq to map chromosomal regions with low/minimal transcription.

**Fig 3:**
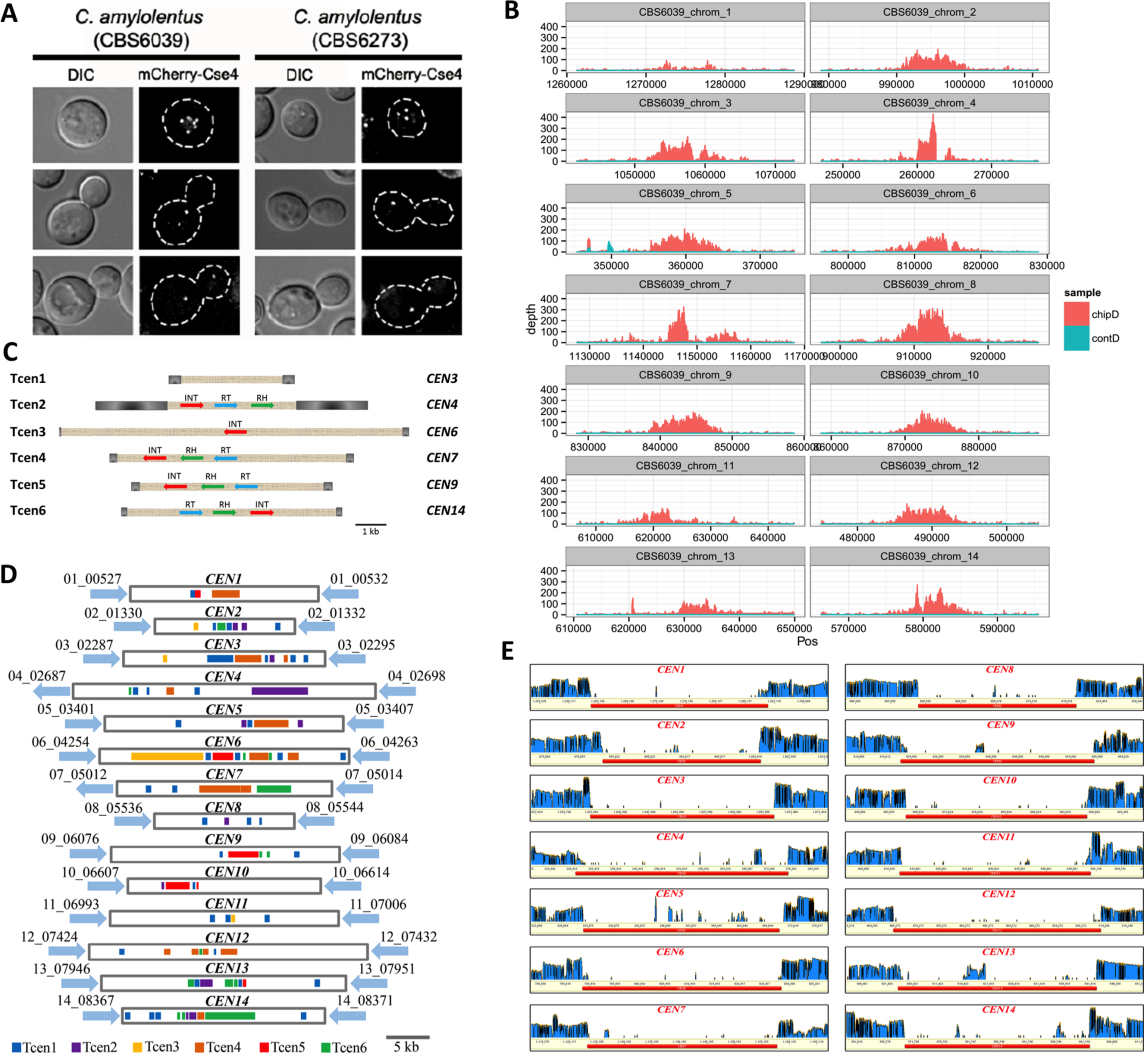
Identification and characterization of centromeres on each of the 14 chromosomes in the CBS6039 genome. (A) Live cell direct fluorescence microscopy images of centromere binding protein Cse4 (CENP-A) at 3 different stages of the mitotic cycle. (B) Plots of read depths when mCherry-CENP-A chromatin immunoprecipitation sequencing (ChIP-seq) data were mapped against the CBS6039 genome assembly are presented. All of the centromeric regions identified in the CBS6039 genome (except for chromosome 1; see Results for more details) showed significantly higher read depth when compared to flanking non-centromeric regions (see S3 Fig for plots of whole chromosomes). Red plots (chipD) are based on signals obtained from ChIP-seq analysis, while blue plots (contD) indicate the negative control. (C) The diagram depicts the structures of the 6 unique centromere-specific Long Terminal Repeat (LTR) retrotransposons, Tcen (transposons in centromeres) 1–6, identified in the *Cryptococcus amylolentus* centromeric regions. While Tcen1 contains only LTRs (shown in grey), all of the other 5 Tcen elements consist of various genes/domains found in retrotransposons (RH, RNaseH; RT, Reverse Transcriptase; INT, Integrase). On the far right are the corresponding centromeres in the CBS6039 genome within which the full-length Tcen elements have been identified. (D) Schematic illustrating the distributions of the 6 Tcen elements, as well as their remnants, on the identified centromere regions in the CBS6039 genome. These intervals were defined as the longest ORF-free regions on the respective chromosomes and contain mostly retrotransposons or their remnants, and show enrichment of CENP-A binding based on ChIP-seq analyses. (E) RNA sequencing (RNA-seq) analysis reveals that the identified CBS6039 centromere regions also had reduced levels of transcriptional activity when compared to flanking non-centromeric regions. The blue bars indicate RNA-seq read depth. Please see S3 Table for coordinates of the centromeres in *C*. *amylolentus*.

CENP-A is the centromere-specific histone H3 variant and has been widely used to identify centromeres [19]. First, we performed CENP-A ChIP-seq analysis using strains derived from CBS6039 where CENP-A, a conserved kinetochore protein, was tagged with mCherry. Specifically, we randomly inserted a genetic construct expressing an mCherry-CENP-A fusion protein into both the CBS6039 and CBS6273 genomes. Live cell imaging at different stages of mitosis revealed that the mCherry-tagged CENP-A localized as multiple puncta in unbudded cells and was seen as single puncta in dividing cells. These mitotic localization patterns of CENP-A in *C*. *amylolentus* are consistent with those observed in the closely related species *C*. *neoformans*, with the centromeres coalescing to form a single cluster as cells progress toward mitosis (Fig 3A), suggesting that the inserted mCherry-CENP-A allele is functional [23]. ChIP pull-down using anti-mCherry (CENP-A) antibodies and subsequent sequencing of the ChIP DNA was performed using the Illumina platform for the CBS6039 transformant. ChIP-seq reads were then mapped back onto the CBS6039 genome. In most cases only 1 region of approximately 7–10 kb in length was found to be significantly enriched (>100-fold) on each of the 14 chromosomes, suggesting that these regions are likely centromere regions on their respective chromosomes (Fig 3B and S3 Fig). We observed a region with only modestly enriched ChIP-seq reads on CBS6039 chromosome 1. This is likely due to the fact that the chromosome 1 scaffold was joined together from 2 individual scaffolds based on evidence from chromoblot analysis and the presence of repetitive elements at one end of each of the 2 initial scaffolds. Thus, it is possible that the centromere DNA sequence of chromosome 1 may be incomplete in our current assembly of CBS6039. We also observed an additional minor CENP-A enrichment peak on chromosomes 9 and 10 (S3 Fig). However, the minor peak on chromosome 9 was located at the end of the chromosome, likely in the telomeric region. Additionally, in both cases the minor peaks overlapped with signal enrichments in the reads of total DNA controls, suggesting these peaks are likely false positives due to unique features of those chromosomal regions (e.g., telomeric repeats). Thus, our ChIP-seq data provide evidence that the centromeres in *C*. *amylolentus* are regional, similar to those in closely related species within the human pathogenic *Cryptococcus* species complex [19,24].

We previously demonstrated that centromeres of the pathogenic *Cryptococcus* species complex are relatively free of ORFs and are enriched with repetitive sequences, transposable elements, and their remnants [19]. We hypothesized that centromeres in *C*. *amylolentus* would share similar features with those of the pathogenic species. Additionally, it has been shown in *C*. *neoformans* that only a part of the long ORF-free region on which a centromere lies shows significant enrichment of CENP-A in ChIP-seq analysis [19,24]. Thus, to further define the *C*. *amylolentus* centromeric loci, we pursued a second approach to identify the longest region on each of the chromosomes in the CBS6039 genome that is ORF-free and contains mostly retrotransposons or their remnants (Fig 3C and 3D). One such region was identified on each of the 14 chromosomes in the CBS6039 genome. The length of these bioinformatically predicted centromeric regions ranged between 22,371 and 48,379 bp, and their locations overlapped with the chromosomal regions that showed the most significant enrichments in the ChIP-seq analysis, strengthening the assignment of these regions as centromeres (Fig 3D; S3 Table).

We also identified 6 different retrotransposons in the CBS6039 genome that are specific for these centromeric regions, which are named as transposons in centromeres 1–6 (Tcen1–Tcen6; Fig 3C). While Tcen1 contains only Long Terminal Repeats (LTRs), all of the other 5 Tcen elements (Tcen2–Tcen6) contain genes typically found in retrotransposons, such as those encoding RNaseH, reverse transcriptase, and integrase (Fig 3C). Additionally, each of these 6 Tcen elements could be found in an apparently complete sequence in at least 1 centromere (Fig 3C). Moreover, all of the centromeres contained multiple additional fragments of different Tcen elements (Fig 3D).

Our third approach, the RNA-seq analysis of the CBS6039 transcriptome, revealed the absence of poly(A) RNA from all 14 centromeric regions identified in the CBS6039 genome (Fig 3E). This is consistent with the relative absence of RNA PolII-mediated transcription found in the regional centromeres of the pathogenic *Cryptococcus* species [19,24].

Because the regions identified by all 3 methods converged to the same region on each of the 14 chromosomes, we conclude that these are bona fide centromere regions on each chromosome in CBS6039. Each of these regions is the binding site of the centromeric histone CENP-A, is depleted of ORFs and enriched with retrotransposons and their remnants, and shows significantly reduced levels of transcription (Fig 3 and S3 Fig; S3 Table). All of these features are consistent with the characteristics of regional centromeres and analogous to the *CEN*s that are found in the closely related human pathogenic *Cryptococcus* species [19,24].

### Centromere-mediated chromosomal translocations occurred during the evolution of *C*. *amylolentus* and its closely related species

**Fig 4:**
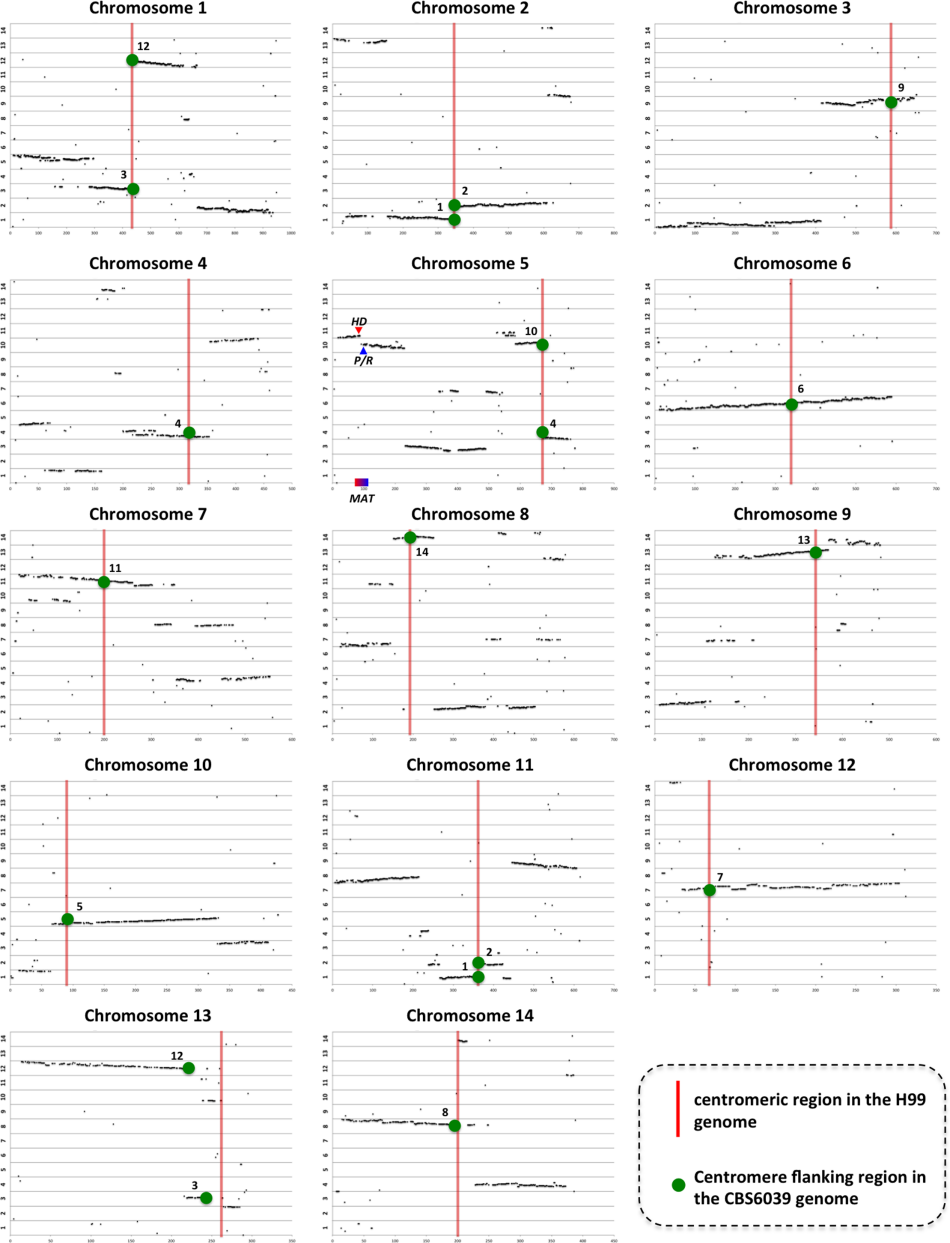
Genome comparison between *Cryptococcus amylolentus* strain CBS6039 and *Cryptococcus neoformans* strain H99. Shown here are distributions of BLAST hits in the CBS6039 genome, using protein sequences of the ORFs from each of the 14 chromosomes in the H99 genome as query. The x-axis shows the numerical order of the ORFs on each H99 chromosome; the y-axis illustrates the 14 chromosomes in the CBS6039 genome. The red vertical bars indicate locations of the centromeres in the H99 genome, the green dots indicate the presence of BLAST hits that are centromere-flanking in the CBS6039 genome, and the numbers beside the green dots indicate the CBS6039 chromosomes from which the centromere-flanking hits are located (see Results and S4 Fig for further details).

Both our CHEF analyses and genome sequencing and assembly indicated significant karyotypic changes between *C*. *amylolentus* and its closely related species within the human pathogenic *Cryptococcus* species complex. To gain a better understanding of the chromosome structural variations that have occurred during the evolution of these 2 lineages, we performed a dot plot analysis of synteny between *C*. *amylolentus* strain CBS6039 and *C*. *neoformans* strain H99 (Fig 4). Our analyses revealed that while large syntenic blocks have been conserved, extensive chromosomal rearrangements are present between the 2 genomes, including both intrachromosomal changes (e.g., inversions and transpositions) as well as interchromosomal translocations. Specifically, with the sole exception of chromosome 6 in H99, which is the homolog of chromosome 6 in CBS6039, all of the other chromosomes in H99 are composed of synteny blocks of varying sizes in the CBS6039 genome, with transpositions and inversions found within most of the syntenic blocks (Fig 4 and S4 Fig).

**Fig 5:**
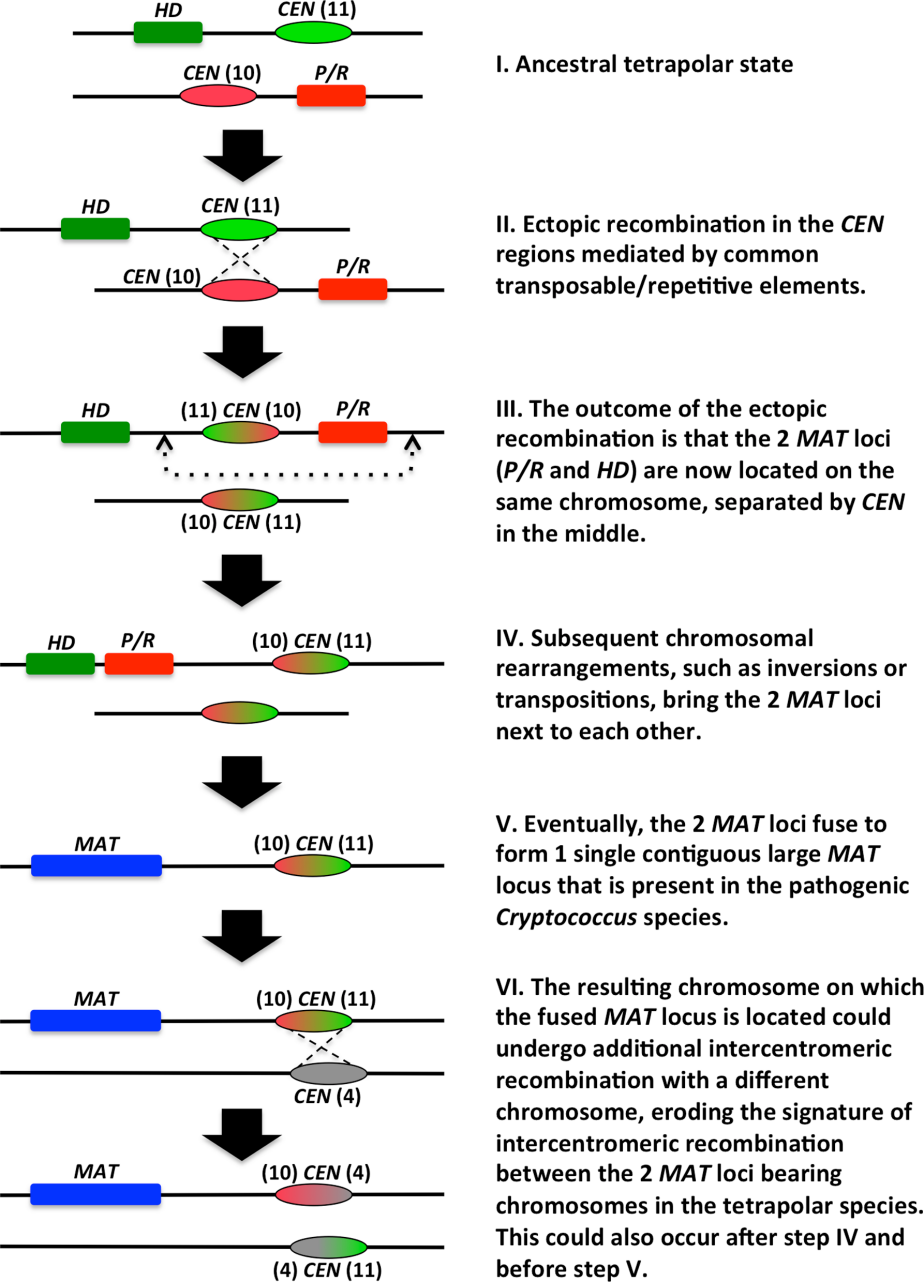
Model for the transition from tetrapolar to bipolar mating system organization. (I) In the ancestor, the *P/R* and *HD* loci were located on different chromosomes, which had regional centromeres that shared common transposable/repetitive elements. (II) and (III) Ectopic recombination occurred between the 2 chromosomes within the centromeric regions, possibly mediated by the common transposable/repetitive elements, bringing the 2 mating type (*MAT*) loci onto the same chromosome. (IV) Subsequent chromosomal rearrangements (e.g., inversions and transpositions) bring the 2 *MAT* loci next to each other. (V) Eventually the *P/R* and *HD* loci fuse to form a single contiguous *MAT* locus that is present in the derived bipolar mating system. (VI) The resulting chromosome with the contiguous *MAT* locus could undergo additional intercentromeric recombination events. The numbers in the parentheses next to the “*CEN*” indicate the *C*. *amylolentus* chromosome on which those centromeric flanking regions are located.

However, despite the vast number of chromosomal translocations that have occurred between the 2 species, the centromere-flanking regions have been largely maintained between the 2 genomes. Specifically, with the exception of chromosome 13 in the H99 genome, the centromeres of all of the other 13 chromosomes in the H99 genome are immediately flanked by genes that are also found to be centromere-flanking in the CBS6039 genome (Fig 4 and S3 Table). Interestingly, for 5 of the H99 chromosomes (ch.1, ch.2, ch.5, ch.11, and ch.14), the 2 flanking regions of the centromeres lie in the flanking regions of different chromosomes in the CBS6039 genome. For example, both H99 chromosomes 2 and 11 are flanked by centromere-flanking regions of CBS6039 chromosome 1 on the left and of CBS6039 chromosome 2 on the right, which can best be explained by a chromosomal arm exchange achieved via ectopic recombination within the 2 centromeric regions that are flanked by these centromere-flanking regions to result in a balanced chromosomal translocation. Importantly, this analysis reveals that *C*. *neoformans* chromosome 5 harboring the *MAT* locus has a centromere derived from intercentromeric recombination mediated translocation involving 2 of the *C*. *amylolentus* chromosomes. As presented in the Discussion (see also Fig 5 and S4 Fig), this leads to a model for the evolution of the linked bipolar *MAT* configuration from the ancestral tetrapolar state.

Thus, our analyses revealed that while the centromeric regions typically undergo accelerated evolution, the centromere-flanking regions can be relatively stable and maintained between different species. Additionally, the centromeres can play important roles in the speciation process, such as by harboring common shared transposable elements and thereby mediating ectopic recombination that likely introduced dramatic karyotypic changes (see Fig 5, S4 Fig, and Discussion), which could have facilitated the establishment and reinforcement of species boundaries.

### Tetrad analyses reveal genetic linkage of both *P/R* and *HD MAT* loci to their respective centromeres in *C*. *amylolentus*

From our analysis of centromeres in the *C*. *amylolentus* genome, we found that the distances between the *P/R* and *HD* loci and the centromeres of their host chromosomes are approximately 146 kb and 435 kb, respectively. The relatively large physical distances between the 2 *MAT* loci and the centromeres of their host chromosomes suggest the *MAT* alleles should undergo random assortment during meiosis. However, when we analyzed the mating types of the progeny dissected from individual basidia, we found that for the *P/R* and *HD* loci, the frequencies of tetratype (TT) basidia were significantly lower than expected when compared to those of parental ditype (PD) and non-parental ditype (NPD) basidia, indicating that both *MAT* loci and centromeres are linked.

Specifically, we dissected a total of 76 basidia from crosses between strains CBS6039 and CBS6273, as well as from crosses between mating compatible F1 progeny (S4 Table). The spore germination rates ranged from 18% to 100%, with an average germination rate of 55%. Additionally, the basidia dissected from F1 intercrosses had a higher average germination rate (64%) compared to basidia dissected from crosses between CBS6039 and CBS6273, which had an average germination rate of 50% (S4 Table; Student *t* test, *P* < 0.05).

We genotyped each of the meiotic progeny from the 76 basidia that were dissected, using the *STE3* (Chrom10_17) and *SXI2* (Chrom11_08) genes as markers for the *P/R* and *HD* loci, respectively (S5 Table). We then determined the tetrad-type of each basidium with respect to the *P/R* and *HD* loci. In cases where the genotyping data of the 2 *MAT* loci were not sufficient to determine the tetrad type of the basidium, we additionally applied markers from both chromosomes 10 and 11, as well as marker S2-2 that is located on chromosome 2 (S5 Table). For example, 5 progeny germinated from basidium No. 1, and all 5 typed as A1B1 for their mating types. However, after we genotyped these progeny with additional markers, we found that there were 2 different genotypes among these 5 progeny when all of the markers are considered, suggesting the other 2 missing genotypes from this basidium all had the A2B2 genotype at the *MAT* loci, and consequently the tetrad type of basidium No.1 was PD.

We were able to unambiguously determine the tetrad types regarding the 2 *MAT* loci for 50 of the 76 basidia. Among these 50 basidia, the ratio of PD, NPD, and TT tetrads was PD:NPD:TT = 11:12:27. The frequency of TT tetrads was significantly lower than expected if neither the *P/R* nor the *HD* loci were to be genetically linked to their respective centromere, in which case the ratio is expected to be PD:NPD:TT = 1:1:4 (Binomial probabilities test, *P* < 0.05). Additionally, while the basidia dissected from F1 intercrosses had higher overall germination rates, the ratios among the 3 types of tetrad (PD:NPD:TT) were similar between basidia dissected from crosses between CBS6039 and CBS6273 (7:6:16) and basidia dissected from F1 intercrosses (4:6:11) (χ^2^ test, *P* > 0.05).

Taken together, our analyses showed that while the physical distances between both the *P/R* and *HD* loci and their respective centromeres are relatively large, there is apparent centromeric linkage for both of the *MAT* loci, which could be due to either reduced recombination in the genome within the *MAT*-*CEN* regions during meiosis, or to deleterious consequences of recombination occurring in these *MAT-CEN* regions that resulted in progeny with reduced fitness that are consequently underrepresented in the progeny descending from germinated meiotic spores.

### Meiotic recombination occurs during sexual reproduction in *C*. *amylolentus* and is comparable with *C*. *neoformans*

It has been shown in a previous study that recombination and chromosomal segregation occur during sexual reproduction in *C*. *amylolentus* [22]. However, the details of the recombination during meiosis, including its nature as well as at what frequency it occurs, had not yet been characterized.

**Fig 6:**
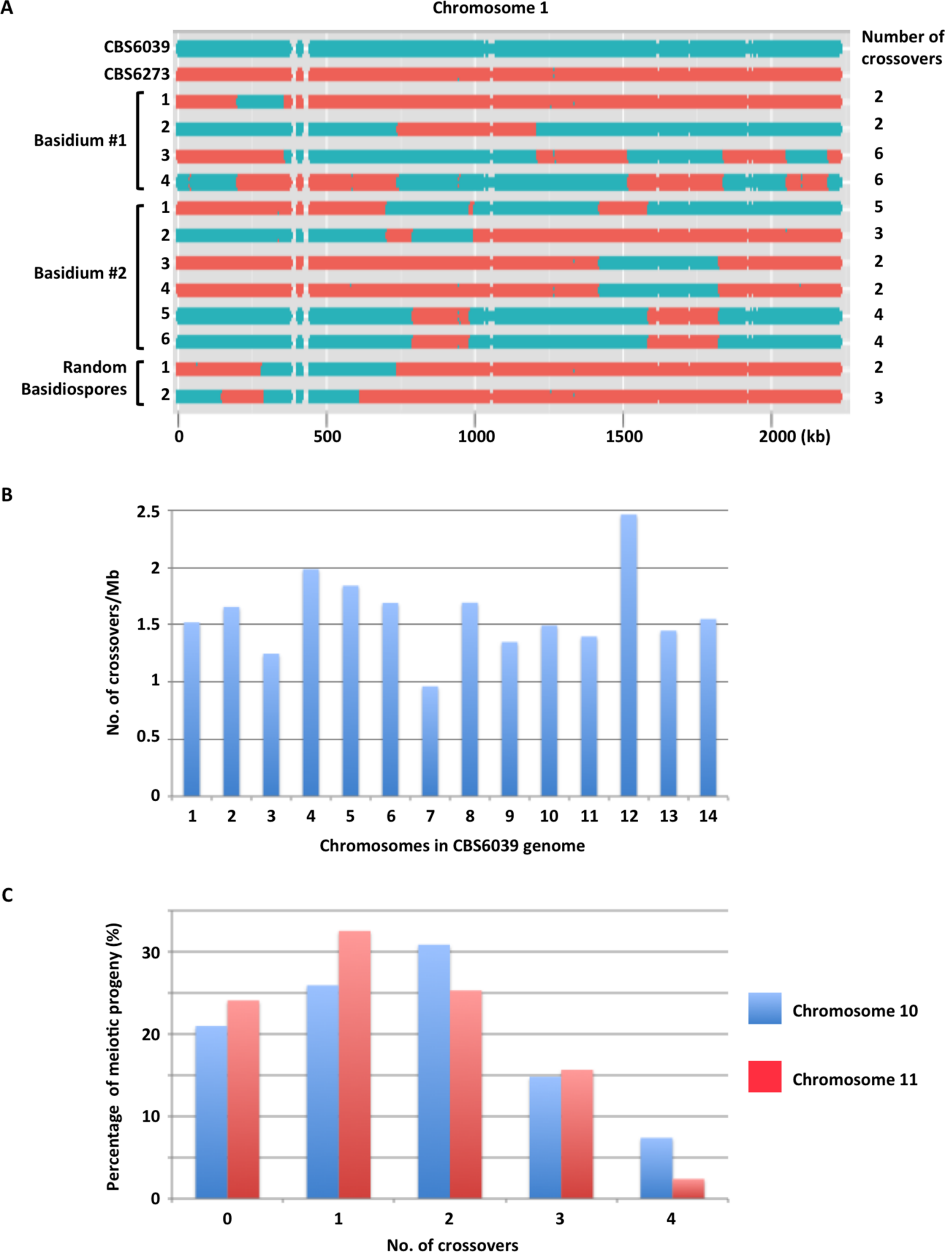
Distribution and frequency of crossovers during sexual reproduction in *C*. *amylolentus*. (A) The SNP distribution along chromosome 1 in meiotic progeny from a cross between strains CBS6039 and CBS6273 suggests that 1 meiotic event occurs per basidium during sexual reproduction in *C*. *amylolentus*. Blue color indicates SNPs that correspond to the genomic sequence of strain CBS6039, and red color indicates SNPs that correspond to the genomic sequence of strain CBS6273. Meiotic progeny from 2 individual basidia (#1 and #2), as well as 2 random basidiospores, were analyzed. For basidium #2, 2 additional basidiospores, #4 and #6, that are genetically identical to basidiospores #3 and #5, respectively, were also included. On the right are the number of estimated crossovers that occurred along chromosome 1 during meiosis in each progeny. (B) The frequencies of crossovers along each chromosome. The data are summarized from S5 Fig. (C) Percentage of progeny that had different numbers of crossovers along chromosomes 10 and 11. Data used to generate the figures can be found at NCBI BioProject with accession no. PRJNA200571 and at EBI with study accession no. PRJEB19939.

To gain further insight into meiotic recombination in *C*. *amylolentus*, we first conducted whole genome sequencing of 10 meiotic progeny recovered from crosses between strains CBS6039 and CBS6273, including 4 progeny from 1 NPD basidium, 4 progeny from 1 TT basidium, as well as 2 random basidiospores. By mapping the sequences onto the CBS6039 genome, we generated plots for the distributions of SNPs between CBS6039 and CBS6273 on each chromosome for each F1 meiotic progeny (Fig 6A and S5 Fig). Based on this analysis, crossovers could be readily scored as transitions between haplotype blocks from the 2 parental strains along the chromosomes.

We found that when unambiguous SNPs were considered, the alleles among the 4 meiotic progeny from the same basidium were always balanced, with a ratio of 2:2 between the alleles from the 2 parents, which suggests that (1) there was only 1 meiotic event that occurred in each basidium during sexual reproduction of *C*. *amylolentus*, and (2) the reshuffling of genetic material during sexual reproduction was mostly through crossovers. Additionally, we calculated the frequency of crossovers (number of crossovers/Mb) along each chromosome and found that the ratio ranged between 0.96 (chromosome 7) and 2.46 (chromosome 12), with an average of 1.59 crossovers/Mb across all of the 14 chromosomes (Fig 6B).

We next focused on the two chromosomes, 10 and 11, on which the *P/R* and *HD* loci reside, respectively. We developed 29 codominant genetic markers located along chromosome 10, and 25 codominant genetic markers located along chromosome 11. Among the 29 markers along chromosome 10, 5 (Chrom10_13 to Chrom10_17) are located within the *P/R* locus, 1 (Chrom10_21) is located within the *CEN* region, and 1 (Chrom10_21) is located within the *CEN*-flanking region. For markers on chromosome 11, 1 (Chrom11_08) is located within the *HD* locus, and 2 (Chrom11_16 and Chrom11_17) flank the centromere of chromosome 11 (S5 Table).

We applied these genetic markers to genotype 84 meiotic progeny collected from 30 basidia. For both chromosomes 10 and 11, all of the 30 basidia had spores corresponding to 1 to 4 genotypes, consistent with a single meiotic event in each. We found 3 progeny (A022, A059, and A831) for chromosome 10, and 3 progeny (A022, A537, and A770) for chromosome 11 that had one or more loci that were heterozygous, indicating chromosomes 10 and 11 were likely disomic in these progeny.

Among the progeny that did not show heterozygosity at any locus, the vast majority had 1 to 4 crossovers along chromosomes 10 (79%) and 11 (76%), with more than half of the progeny (57% for chromosome 10 and 58% for chromosome 11) having 1 or 2 crossovers (Fig 6C). On the other hand, there was no evidence of recombination on chromosome 10 in 21% of the progeny, and on chromosome 11 in 24% of the progeny (Fig 6C). However, it is still possible that recombination did occur on chromosomes 10 and 11 in these progeny, but in regions not covered by the genetic markers analyzed, such as subtelomeric regions.

In summary, our results show clear evidence that recombination occurs during sexual reproduction in *C*. *amylolentus*, with the majority of the chromosomes having crossovers at a frequency between 1 to 2 crossovers/Mb, and the majority of the progeny having anywhere between 1 and 4 crossovers along chromosomes 10 and 11, respectively.

### Regions between the *MAT* loci and their respective centromeres show reduced recombination frequencies and form linkage disequilibrium blocks

We next constructed genetic linkage maps for the 29 markers on chromosome 10 and the 25 markers on chromosome 11. For each chromosome we utilized the 81 mating products that were monomorphic at all of the genetic markers on that particular chromosome.

**Fig 7:**
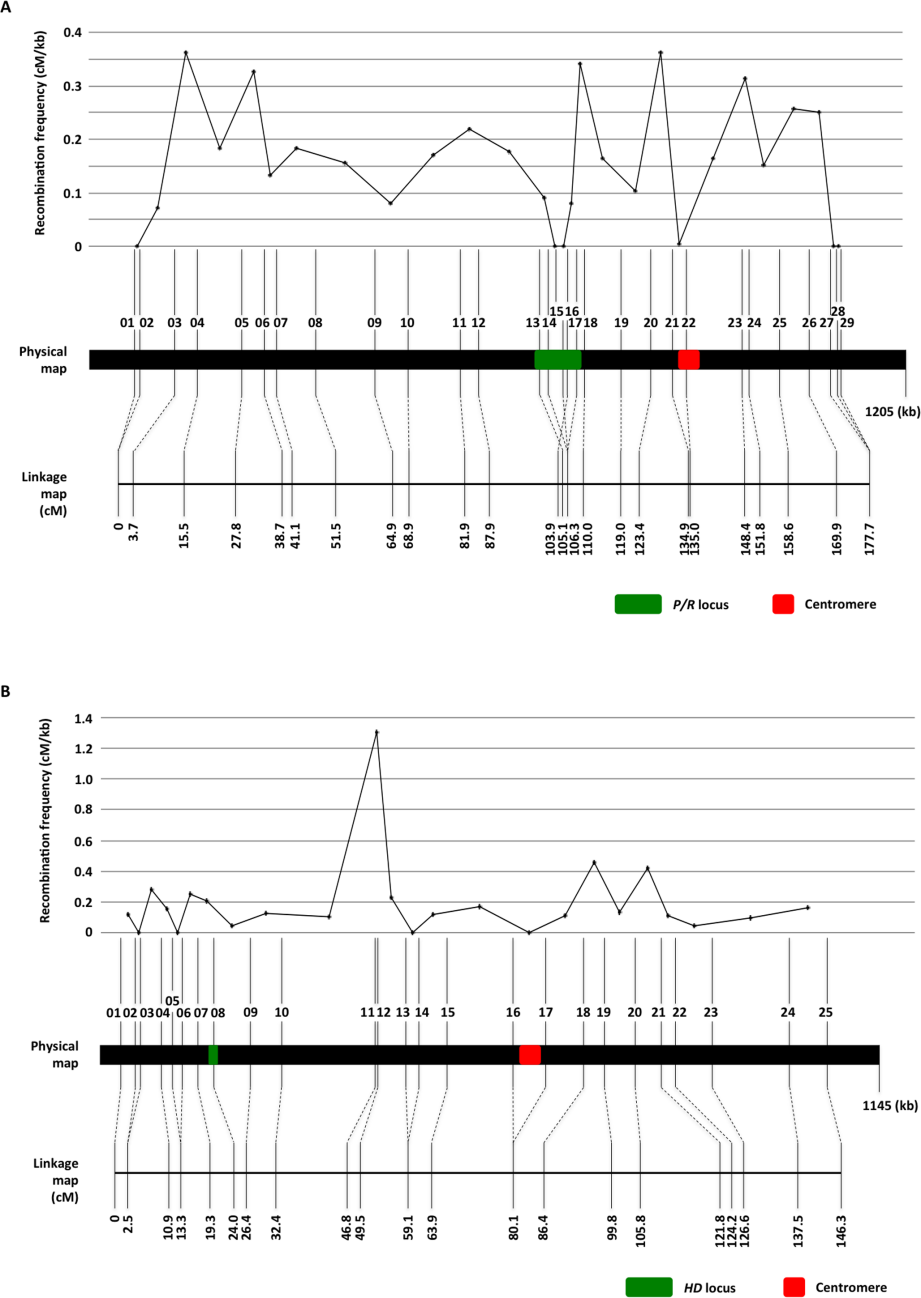
Meiotic recombination frequencies observed on chromosomes 10 and 11. For both chromosomes 10 (A) and 11 (B), the top panel shows the recombination frequencies (cM/kb) at different locations along the chromosome, calculated based on the physical locations of the genetic markers on the chromosome (middle panel; see S5 Table for detailed information on the locations of the markers) and the genetic distances between markers that were estimated from the genetic linkage map (bottom panel). The green and red blocks indicate the locations of the mating type (*MAT*) loci (the *P/R* locus on chromosome 10 and the *HD* locus on chromosome 11) and the centromeres, respectively.

The 29 markers from chromosome 10 formed one linkage group (Fig 7A). The order of markers within the linkage group was in overall agreement with their physical positions on chromosome 10, with the exception of 1 marker (Chrom10_16) that was located within the *P/R* locus. The chromosome 10 linkage group was 177.7 cM in size and encompassed 1041 kb of chromosome 10, which produced an average recombination frequency of 5.86 kb/cM (0.17 cM/kb). There were 6 marker intervals within which no genetic distance was detected, of which 3 were in between genetic markers that were located less than 20 kb from each other (intervals 01–02, 27–28, and 28–29 in Fig 7A); 2 were located within the *P/R* locus (intervals 14–15 and 15–17) and 1 (interval 21–22) was located in a region that overlapped with part of the centromere (Fig 7A, top panel). On the other hand, we also identified several marker intervals that had high recombination frequencies. Of the 3 intervals that showed the highest recombination frequencies, 1 was located between markers Chrom10_03 and Chrom10_04, 1 was located between markers Chrom10_17 and Chrom10_18 that flank the *P/R* locus, and the last one was located between markers Chrom10_20 and Chrom10_21 that flank the centromere. The reason for the high recombination frequencies observed within these regions is not clear, although it has been shown in previous studies that in *C*. *neoformans*, which is closely related to *C*. *amylolentus*, recombination hot spots also flank the *MAT* locus [25,26]. Additionally, the region encompassing the *P/R* locus and the centromere (interval 13–22) had a recombination frequency of 7.25 kb/cM (0.14 cM/kb), which was lower compared to the average recombination frequency across the linkage group (0.17 cM/kb), as well as the 2 regions located outside of the *MAT*-*CEN* region on chromosome 10: marker interval 1–13 (0.15 cM/kb) and marker interval 22–29 (0.19 cM/kb).

The 25 markers from chromosome 11 also formed one linkage group (Fig 7B) and the order of markers within the linkage group was in agreement with their physical positions on chromosome 11. The chromosome 11 linkage group was 146.3 cM in size and encompassed 1,041 kb of chromosome 11, which produced an average recombination frequency of 7.12 kb/cM (0.14 cM/kb). There were 4 marker intervals within which no genetic distance was detected, of which 1 (interval 16–17) was located in a region that encompassed the centromere. There was also 1 marker interval (11–12) that was located between the *HD* locus and the centromere that showed a significantly higher recombination frequency, which could indicate the presence of a recombination hot spot in that region. However, despite the presence of this potential recombination hot spot, the region between the *HD* locus and centromere (interval 8–16) had an average recombination frequency of 7.81 kb/cM (0.13 cM/kb), which was slightly lower when compared to the average recombination frequency of the chromosome 11 linkage group (0.14 cM/kb), as well as the 2 regions located outside of the *MAT*-*CEN* region on chromosome 11: marker interval 1–8 (0.14 cM/kb) and marker interval 17–25 (0.16 cM/kb).

Consistent with the slightly reduced recombination frequencies observed in regions between the *MAT* loci and their respective centromeres, when we analyzed the genotyping data for the presence of linkage disequilibrium (LD) between markers, we identified the presence of LD blocks on both chromosomes 10 and 11 that encompassed the *MAT* loci and their centromeres, within which marker pairs with highly skewed allele combinations were observed (S6 Fig).

Taken together, we found that the regions between the *MAT* loci and their respective centromeres show reduced, but not completely suppressed recombination during meiosis in *C*. *amylolentus*, which could explain the observed under-representation of TT tetrads. The identification of LD blocks and non-random allele associations among markers in these regions also suggest the possible existence of coadapted alleles within these chromosomal regions, for which recombination could break up the favorable allele combinations and consequently result in progeny with reduced fitness.

## Discussion

The genome assemblies of the 2 *C*. *amylolentus* isolates, CBS6039 and CBS6273, are both approximately 20.3 Mb in size, which are comparable to those of the species within the closely related pathogenic *Cryptococcus* species complex [27]. Compared to pathogenic *Cryptococcus* species, 4 pathways appeared to have undergone significant expansion in the *C*. *amylolentus* genome, which could be the result of different selection pressures that the 2 groups have experienced during their evolution.

It has been shown in previous studies that *C*. *amylolentus* has a tetrapolar mating system with the 2 *MAT* loci located on different chromosomes and undergoing random assortment during meiosis [22]. This is different from species in the pathogenic *Cryptococcus* species complex that have bipolar mating systems with only 1 biallelic *MAT* locus. In a previous study, we showed that the *MAT* loci in *C*. *amylolentus* have already undergone expansion, based on the distributions in the *C*. *amylolentus* genome of the genes located within the *MAT* locus in the pathogenic *Cryptococcus* species complex. However, due to the limited sequence information, especially the lack of extensive sequence for the *MAT* loci of *C*. *amylolentus* strain CBS6273, the boundaries of the *P/R* and *HD* loci in *C*. *amylolentus* were not clearly identified. In this study, we obtained the complete sequences of the 2 chromosomes (10 and 11) on which the *P/R* and *HD* loci are located, respectively, from both *C*. *amylolentus* strains CBS6039 and CBS6273. Because these 2 strains are mating compatible, they should have different *MAT* alleles with elevated sequence divergence and/or chromosomal rearrangements at both of the *MAT* loci. By comparing the sequences between CBS6039 and CBS6273, we indeed identified 2 chromosomal regions on chromosomes 10 and 11 that showed significant divergence between the 2 isolates and were flanked by the chromosomal regions that showed complete synteny between the 2 *C*. *amylolentus* isolates. We defined the 2 divergent regions as the *P/R* and *HD* loci in *C*. *amylolentus*, respectively (Fig 2 and S2 Fig; see Results). The *P/R* and *HD* loci in *C*. *amylolentus* are approximately 96 kb and 22 kb (or 5 kb if only the *SXI1* and *SXI2* genes are included) in size, respectively, which sums to a size similar to that of the *MAT* locus (120 kb) in the pathogenic *Cryptococcus* species [28]. Additionally, the *C*. *amylolentus P/R* and *HD* loci encompassed the majority of the genes located within the *MAT* locus of the pathogenic *Cryptococcus* species, with a few species-specific *MAT* genes observed in both *C*. *amylolentus* and pathogenic *Cryptococcus* species (e.g., the *RPL22*, *SPO14*, and *STE11* genes in pathogenic *Cryptococcus* species; Fig 2 and S2 Fig), suggesting there has been a relatively limited evolutionary history since the transition from the tetrapolar mating system present in the common ancestor of *C*. *amylolentus* and pathogenic *Cryptococcus* species to the bipolar mating systems that are retained in the pathogenic *Cryptococcus* species.

Studies have shown that genes located within the *MAT* locus of pathogenic *Cryptococcus* species have had different evolutionary histories, with significantly lower levels of divergence observed in some genes compared to others [29]. It has also been hypothesized that a gene cluster encompassing the *GEF1*, *CID1*, *LPD1*, *BSP2*, and *RPO41* genes that all showed low divergence among pathogenic *Cryptococcus* species was recruited into the *MAT* locus recently, maybe coincident with and possibly even mediating the fusion of the *P/R* and *HD* loci that gave rise to the single *MAT* locus in the pathogenic *Cryptococcus* species [22,29–31]. Based on our analysis of the complete *P/R* and *HD* loci, these 5 genes are all located within the *P/R* locus of *C*. *amylolentus*. Additionally, these genes no longer form a single cluster and have undergone chromosomal rearrangements between the 2 *C*. *amylolentus* strains (Fig 2 and S2 Fig). Thus, it is likely that these genes were already located within the *P/R* locus in the common ancestor of *C*. *amylolentus* and the pathogenic *Cryptococcus* species, and the fusion of the ancestral *P/R* and *HD* loci that gave rise to the bipolar mating system was initiated by ectopic recombination mediated by other genomic elements, such as transposable elements and repetitive sequences. For example, it has been shown that in the yeast *Saccharomyces cerevisiae*, chromosomal translocations could be generated by high frequency meiotic recombination between repeated genes [9], or by mitotic recombination mediated by retrotransposons (Ty elements) under certain conditions [8].

Both *C*. *amylolentus* and the pathogenic *Cryptococcus* species have regional centromeres that are enriched with transposable elements and repetitive sequences, with some elements shared among different chromosomes [19,27] (also see Results). This provides opportunities for ectopic recombination to occur within the centromeres and between different chromosomes. It has been shown previously that chromosomal arm exchange mediated by the centromeres contributed to the genetic divergence among species within the pathogenic *Cryptococcus* species complex [19]. A recent study showed that similar centromere-mediated chromosomal rearrangements could have also occurred during the evolution of the ascomycetous budding yeast *Candida tropicalis* that also possesses repeat-associated regional centromeres [20]. Additionally, several lines of evidence from recent studies challenge the dogma that centromeres are typically recombination-deficient regions. Specifically, it has been shown that recombination occurs at frequencies higher than expected in centromeres ranging from the point centromere in yeast to regional centromeres in plants and animals, resulting in gene conversion or chromosomal translocations [15–18].

We propose a model in which the inciting event for the transition from the tetrapolar mating system to the bipolar mating system was mediated by ectopic recombination between the centromeric regions of the 2 chromosomes on which the *P/R* and *HD* loci reside. The outcome of such a chromosomal rearrangement is that the 2 *MAT* loci are now located on the same chromosome, but lying on opposite sides of the centromere. Additionally, as we showed in *C*. *amylolentus*, if both the *P/R* and *HD* loci are centromere linked, this could have facilitated translocation events that established linkage between the *P/R* and *HD* loci, reducing recombination between the 2 loci and mirroring the *MAT* configurations observed in species with pseudobipolar mating systems. Subsequent chromosomal rearrangements, such as inversions and transpositions, could bring the 2 *MAT* loci closer to each other and eventually result in the complete linkage between the 2 *MAT* loci to form the single contiguous *MAT* locus in the extant bipolar mating system (Fig 5 and S4 Fig). This model is consistent with the findings from our study, as well as previous studies of the *MAT* locus in other fungal species [4,6,32]. It should be noted that the centromere of the *C*. *neoformans* chromosome 5, on which its *MAT* locus is located, is not flanked by the centromere-flanking sequences from *C*. *amylolentus* chromosomes 10 and 11, on which the *P/R* and *HD* loci are located, respectively. Instead, the *C*. *neoformans* chromosome 5 centromere is flanked by a centromere-flanking region from chromosome 10 on one side, and a centromere-flanking sequence from *C*. *amylolentus* chromosome 4 on the other. One possible explanation could be that after the initial ectopic intercentromeric recombination that brought the 2 *MAT* loci onto the same chromosome and the subsequent chromosomal rearrangement that brought the 2 *MAT* loci into close proximity, the resulting chromosome underwent additional intercentromeric ectopic recombination with another chromosome (e.g., *C*. *amylolentus* chromosome 4; Fig 5 and S4 Fig). This would result in the extant *C*. *neoformans* chromosome bearing the *MAT* locus being only flanked on one side by the centromere-flanking sequences of *C*. *amylolentus P/R* or *HD* chromosomes. Another possibility is that the chromosomal/centromere organization in *C*. *amylolentus* may not fully reflect those present in the genome of the common ancestor of *C*. *amylolentus* and *C*. *neoformans*, and subsequent chromosomal rearrangements have occurred within each lineage during their descent from their common ancestor. There is also the possibility that the single *MAT* loci in *C*. *neoformans* could be the result of translocation of 1 *MAT* locus of the tetrapolar mating system to the other *MAT* locus. However, our analysis showed that the chromosomal rearrangements between *C*. *neoformans* chromosome 5 and *C*. *amylolentus* chromosomes 10 and 11 encompassed regions that are significantly larger than the *HD* and *P/R* loci. Also, at least for the *P/R* locus (*C*. *amylolentus* chromosome 10), this chromosomal rearrangement breakpoint occurred at the centromere. Additionally, the centromere of *C*. *neoformans* chromosome 5 is flanked by centromere-flanking sequences from different chromosomes in the *C*. *amylolentus* genome (S4 Fig), consistent with it being the result of intercentromeric ectopic recombination. Taken together, while we could not rule out the possibility of the *HD* and *P/R* loci being brought together by simple translocation, we propose that the establishment of linkage between *HD* and *P/R* loci can be best explained by centromere-mediated ectopic recombination followed by intrachromosomal rearrangements that, compared to interchromosomal rearrangements, more readily occur. Also, the karyotypic changes established through intercentromeric ectopic recombination likely resulted in chromosomes that had difficulty in pairing during meiosis, which would further increase the chances of additional chromosomal rearrangements occurring on those chromosomes. Our ongoing efforts in analyzing the genomes and *MAT* loci in species closely related to *C*. *amylolentus* and *C*. *neoformans* will provide further insights into the evolutionary transitions between the tetrapolar and bipolar mating systems.

We found both the *P/R* and *HD MAT* loci in *C*. *amylolentus* exhibit centromeric linkage during sexual reproduction, with the frequencies of TT type tetrad recovered from meiosis significantly lower than expected if the *MAT* loci were not centromere linked. The chromosomal regions encompassing the *MAT* loci and their centromeres also form blocks with significant LD among meiotic progeny (S6 Fig). It should be noted that although the 2 *MAT* loci appeared to be genetically linked to their respective centromeres, the inter–*MAT*-centromere regions did not show significantly enhanced sequence divergence or chromosomal rearrangements compared to other chromosomal regions, and some intervals within these inter–*MAT*-centromere regions showed recombination frequencies that were comparable to other chromosomal regions on chromosomes 10 and 11 (Figs 1 and 7). This suggests that the linkage between the *MAT* loci and their respective centromeres is probably not due to the presence of physical barriers that suppress recombination. One possible alternative explanation could be the presence of genetic elements that are involved in epistatic interactions in these regions, which would favor the co-segregation of alleles from the same parent in these regions, resulting in LD blocks. These epistatic interactions could also involve genetic elements located on different chromosomes, such as those that control the fur pigmentation in the oldfield mice species [33]. However, we did not observe significant LD between markers from chromosomes 10 and 11, including those located close to or within the *MAT* loci.

Our genomic comparison analysis between *C*. *amylolentus* and *C*. *neoformans* strain H99 identified that out of the 14 centromeres, 5 have undergone intercentromeric ectopic recombination resulting in chromosomal arm exchanges during the evolution of the 2 lineages. As mentioned earlier, this type of centromere-mediated ectopic recombination event has also been identified in other species such as *C*. *neoformans* and *C*. *tropicalis* that also possess repeat associated centromeres [19,20]. Thus, intercentromeric ectopic recombination could occur more frequently than currently appreciated. In *C*. *amylolentus* the centromeres coalesce to form a single cluster as cells progress toward mitosis (Fig 3A), which is similar to the observation in pathogenic *Cryptococcus* species [23]. This suggests that physical proximity of centromeres in the cluster could provide opportunities for the centromeres from different chromosomes to interact during cell division and might promote their recombination. Indeed, several recent studies have identified direct interactions between centromeres in a variety of species by Hi-C analysis [34–36]. Thus, it appears that there are ample opportunities for intercentromeric interactions to take place during cell division. It should be noted that this type of intercentromeric ectopic recombination might not occur at a very high frequency due to the significant karyotypic variation that it introduces. However, if it happened and the dramatic karyotypic variation that it induces survived selection, this could in turn facilitate the divergence being established within each of the diverging lineages. Additionally, intercentromeric recombination involving 2 monocentric chromosomes gives rise to 2 derived monocentric chromosomes; in contrast, translocations not mediated by the centromere would yield an acentric and a dicentric chromosome, both of which are mitotically unstable.

The model that we propose is also consistent with the observations of the *MAT* loci organizations in several other basidiomycetous species that have unusual *MAT* structures. Specifically, the *MAT* organizations in *C*. *amylolentus* and *C*. *neoformans* represent the stages I and V–VI in our proposed model (Fig 5), respectively, and there are no known closely related species that have *MAT* loci mirroring the intermediate transition stages III and IV. However, in another basidiomycetous species cluster that includes *Ustilago maydis*, *Ustilago hordei*, and *Ustilago bromivora*, while *U*. *maydis* possesses a classic tetrapolar mating system with the *P/R* and *HD* loci located on different chromosomes, the 2 *MAT* loci are located on the same chromosome in both *U*. *hordei* [4] and *U*. *bromivora* [5]. Interestingly, while the region separating the *P/R* and *HD* loci in *U*. *hordei* is approximately 450–500 kb in size, the distance between the *P/R* and *HD* loci in *U*. *bromivora* is significantly less (approximately 183 kb). Thus, the configurations of the *MAT* loci in *U*. *hordei* and *U*. *bromivora* could represent the different intermediate stages proposed in our model or they could represent independent events that resulted in the physical linkage of the *P/R* and *HD* loci in these 2 species. In particular, the *MAT* chromosome of *U*. *hordei* might harbor the centromere in between the linked a and b loci.

Additionally, in another basidiomycetous species cluster that includes *Malassezia sympodialis*, *Malassezia globosa*, and *Malassezia yamatoensis*, linkage between the *P/R* and *HD* loci has been observed in strains belonging to all 3 species [6]. Again, while the distance between the *P/R* and *HD* loci are similar between *M*. *sympodialis* and *M*. *globosa* (approximately 141 kb and 168 kb, respectively), the chromosomal region between the *P/R* and *HD* loci in *M*. *yamatoensis* is significantly larger (approximately 586 kb). Also, comparing the configurations of the *MAT* loci in the 3 species suggests that 1 of the 2 *MAT* loci underwent inversion between the *M*. *yamatoensis* lineage and the lineage leading to *M*. *sympodialis* and *M*. *globosa*. It has been proposed that the *MAT* locus configuration in the *Malassezia* species represents a “pseudo-bipolar” state, in that the *P/R* and *HD* loci are physically distantly linked with limited recombination in between [7,37]. In our model, the *MAT* loci structures in these *Malassezia* species could actually represent the intermediate stages of the transition from an ancestral tetrapolar mating system to a derived bipolar mating system, although the structures and locations of the centromeres in the species belonging to the *Ustilago* and *Malassezia* species complexes remain to be defined. Thus, characterization of the structures and locations of the centromeres with respect to the *MAT* loci in *Ustilago* and *Malassezia* species will allow further tests of the model that intercentromeric recombination events have facilitated transitions in both genomic organization and *MAT* configuration.

### Conclusion

In this study, we generated high-quality genome assemblies of 2 *C*. *amylolentus* strains, CBS6039 and CBS6273, which are closely related to the human pathogenic *Cryptococcus* species complex. Our mouse experiments confirmed that *C*. *amylolentus* is non-pathogenic. Additionally, in contrast to the bipolar mating system in the pathogenic *Cryptococcus* species, *C*. *amylolentus* has a classical tetrapolar mating system that is ancestral in basidiomycetes. Moreover, the 2 *P/R* and *HD MAT* loci contain almost all of the genes that are present in the *MAT* locus of the bipolar pathogenic *Cryptococcus* species. Based on several lines of evidence, including (1) the genetic linkage of both *P/R* and *HD MAT* loci to their respective centromeres in *C*. *amylolentus*, (2) the regional centromeres in *C*. *amylolentus* are enriched with species-specific transposable elements and repetitive sequences that are shared among *CEN*s, as well as (3) evidence of chromosomal arm exchanges that have occurred after *C*. *amylolentus* and the pathogenic *Cryptococcus* species split from their common ancestor, we propose a model of transition from an ancestral tetrapolar mating system to a derived bipolar mating system that is initiated by intercentromeric ectopic recombination mediated by common transposable/repetitive elements shared between centromeres of different chromosomes. This model is consistent with recent findings of the *MAT* structures in other basidiomycetous species complexes. Our findings lay a foundation for future studies of the evolution of the *MAT* locus, as well as the emergence and evolution of virulence and pathogenicity in pathogenic *Cryptococcus* species, and possibly also other basidiomycetous pathogens. The observation that genomic organization has been reorganized as a result of repeated intercentromeric recombination events leading to chromosomal translocations may turn out to be a more general feature of genome evolution. This type of translocation may occur more readily because, unlike other translocation mechanisms that could lead to unstable dicentric and acentric chromosomes, this mechanism yields 2 stable monocentric chromosomes. These events may also lead to speciation events through enforcement of species boundaries by enabling facile changes in karyotype that can lead to isolation from the ancestral karyotype.

## Materials and methods

### Strains and media

*C*. *amylolentus* strains CBS6039 and CBS6273, as well as their progeny, were grown on YPD solid medium unless specified otherwise. Matings were conducted on V8 (pH = 5) solid medium as previously described [22]. Spores were dissected from individual spore chains as described in previous studies [22,38,39].

### DNA extraction, RNA extraction, genotyping, and CHEF electrophoresis and chromoblot analyses

Genomic DNA and RNA extraction, as well as genetic marker screening and genotyping of the *C*. *amylolentus* parental strains and their progeny were conducted following protocols described in previous studies [19,26,38]. CHEF gel electrophoresis and chromoblot analyses were carried out as described in a previous study [22].

### Genome sequencing, assembly, and annotation

For both strains CBS6039 and CBS6237, genomic DNA was used to construct 2 libraries: a small insert library with median insert sizes of 188 or 164 bases, respectively, for CBS6039 and CBS6237 and a large insert library of median insert size of 2.2 or 2.3 kilobases, respectively, as previously described [40,41]. Each library was sequenced on an Illumina HiSeq 2000 to generate 101 base paired-end reads; 100-fold depth of the small library and 50-fold depth of the large insert library of each strain was assembled using Allpaths [42] version *R47093* (CBS6039) or *R47684* (CBS6237). Assemblies at twice these levels of coverage were also evaluated; however, they included many additional small contigs with little other difference in assembly metrics. Both assemblies were evaluated for even coverage of both libraries and checked for contamination using GAEMR (http://software.broadinstitute.org/software/gaemr/). For CBS6039, additional Roche/454 8 kb mate-paired reads as well as PacBio filtered subreads were used for higher order scaffolding using SSPACE-LongRead v1-1 [43], requiring 5 linking reads (-l 5) and a 200 base gap between scaffolds (-g 200).

Genes were predicted and annotated by combining calls from multiple methods. A training set was generated using Genewise and Genemark [44], and then GlimmerHmm [45], Snap [46], and Augustus [47] were run to generate ab initio gene models. The best gene model at a given locus was selected from these data sets using EVM [48]; conserved genes missing in gene sets were identified using OrthoMCL [49] and combined with the EVM set. Genes matching repetitive elements were then filtered if their coordinates overlapped TransposonPSI (http://transposonpsi.sourceforge.net/) hits (>30% overlap to CDS, e-value 1e-10), repeat Pfam domains, or RepeatRunner [50] proteins.

The gene set of CBS6039 was also updated using RNA-seq data. Reads were quality trimmed, jaccard clipped, and normalized using Trinity version 2.1.1 [51]; the filtered reads were then aligned to the genome using STAR version 2.4.2a [52] with parameter—alignIntronMax 10000. The reads were then assembled into transcripts by providing the STAR aligned bam to Trinity run in the genome-guided mode with parameter—genome_guided_max_intron 10000. Trinity transcripts were aligned to the genome with PASA [48] and provided as input to EVM for gene calling as described above.

### Synteny analysis

Regions of sequence similarity were determined with the NUCmer algorithm from the MUMmer package version 3.23 [53] with maximum gap size set to 10 (—maxgap 10). The results were filtered with the delta-filter algorithm to obtain alignments that form the longest consistent sets for query and reference. The resulting files were used as input to show-coords for analyzing coordinates of aligned regions.

Our identification of the complete *P/R* and *HD* loci in *C*. *amylolentus* also suggests that a previously reported inversion between the *P/R* locus of *C*. *amylolentus* strain CBS6039 and the closely related sibling species *Tsuchiyaea wingfieldii* that involves the contig encompassing the *RPL39* and *MYO2* genes should have been a translocation instead. This mis-assembly of the CBS6039 *P/R* locus was likely due to the fact that the previous assembly of the *P/R* locus was incomplete with 3 separate contigs that were bounded by mating pheromones and repetitive sequences at the junctions.

### Identification of intergenic regions

We scanned the genome of *C*. *amylolentus* (CBS6039) using the Geneious R9 software (http://www.geneious.com) [54] to identify the intergenic regions. The largest intergenic (ORF-free) regions were identified on each chromosome. Some of the predicted ORFs were not considered authentic ORFs because they were either transposon-like or dubious in nature. The ORFs that were smaller than 200 amino acids were also not considered for this analysis.

### Transposon identification and mapping

The LTR-retrotransposons in *C*. *amylolentus* centromeric regions were identified using the LTR-finder program (http://tlife.fudan.edu.cn/ltr_finder/, [55]). Six LTR elements were identified in different centromeres. The sequences of these LTR elements were retrieved from the genome and subjected to sequence analysis for motif/domain analysis using CD-search (http://www.ncbi.nlm.nih.gov/Structure/cdd/wrpsb.cgi). Next, BLASTn analysis was performed to determine occurrence of full length/traces of these elements in the genome, including centromeres. BLAST results were mapped onto the genome and we found that these elements clustered exclusively at the centromeres, and hence these 6 elements were named Tcen1 through Tcen6.

### Construction of *C*. *amylolentus* strains with mCherry-tagged Cse4 (CENP-A)

To tag Cse4 at its N-terminus with mCherry, the promoter region of the *CSE4* ORF (784 bp, primers VYP901 and VYP902), the mCherry gene sequence (708 bp; primers VYP903 and VYP904), and the *CSE4* ORF along with its 3′-UTR as the terminator (1216 bp; primers VYP905 and VYP906) were fused using the overlap PCR method. The promoter, ORF, and terminator regions were amplified from the genomic DNA of *C*. *amylolentus* strain CBS6039, whereas the mCherry sequence was amplified from the pLKB25 plasmid [56]. The full-length PCR product was finally amplified using primers VYP901 and VYP906 containing XbaI and XhoI restriction sites. The amplified product was digested with XbaI and XhoI and cloned into the corresponding sites of pLKB25 to generate pVY50. *C*. *amylolentus* strains CBS6039 and CBS6273 were then transformed with plasmid DNA using biolistic transformation to generate strains SSD502 (CBS6039, mCherry-*CSE4*-*NEO*) and SSD505 (CBS6273, mCherry-*CSE4*-*NEO*), respectively. The transformants were selected on YPD solid medium containing 200 μg/ml G418 and screened for specific mCherry signals using fluorescence microscopy. The desired transformants were grown overnight at 30°C, cells were pelleted and washed with water, and images were captured using a DeltaVision (GE Healthcare) microscope. The images were processed using ImageJ and Adobe Photoshop.

### Identification and characterization of the centromeres by ChIP-seq and RNA-seq assays

ChIP assays were carried out as described previously with minor modifications [19]. Briefly, the mCherry-CENP-A expressing strain (SSD502) was grown in 100 ml YPD liquid medium to OD_600_ = 1. The cells were co-incubated with crosslinker for 30 min, harvested, and resuspended in 10 ml of water containing 0.5 ml of β-ME. The cell suspension was incubated at 30°C for 1 h followed by spheroplasting using the lysing enzyme from *Trichoderma harzianum* (Sigma, Cat. no. L1412). The spheroplasts were resuspended in the lysis buffer and sonicated for chromatin shearing using Bioruptor (Diagenode) for 17 cycles of 15 s on and 15 s off bursts at the high level and fragmented chromatin was isolated. A part of the chromatin fraction (100 μl) was kept for input DNA (I) preparation and the remaining chromatin solution was divided into 2 equal halves (450 μl each). RFP-TRAP beads (Chromotek) were added in one half (+) whereas blocked agarose beads were added in the other tube (-). The tubes were incubated at 4°C for 8 h on a rotator. The beads were then washed and bound chromatin was eluted using elution buffer. All 3 fractions (I, +, and -), were decrosslinked and bound DNA was isolated using phenol:chloroform extraction followed by ethanol precipitation. The precipitated DNA was air dried and dissolved in MilliQ water containing 20 μg/ml RNase (Sigma, Cat. no. R4875). I and + samples were subjected to ChIP-seq to identify CENP-A–rich regions across the CBS6039 genome.

RNA-seq reads were obtained by sequencing the cDNA library with the IlluminaHiSeq 2000 technology (paired-end reads of 2 x101 nucleotides).

The ChIP-seq and RNA-seq reads were aligned to the genome by Geneious R9 software (http://www.geneious.com) [54]. Additionally, the centromere regions with 10 kb flanking on each side were probed for the presence/absence of polyA RNA reads.

### Construction of LD heat maps

To characterize meiotic recombination among genotyped markers on chromosomes 10 and 11, we carried out linkage analysis on all pairwise markers in the F1 population. We estimated the squared correlation coefficient (*r*^2^) between markers i and j using the following equation:

$$r^{2}=\frac{D_{ij}^{2}}{p_{i}(1-p_{i})p_{j}(1-p_{j})}$$

where *p*_*i*_ and *p*_*j*_ are allele frequencies of the 2 markers, and D is defined as the difference between half of the observed frequency of heterozygosity and the expected value, where

$$D=\frac{1}{2}(p_{ij}+p_{ji})-p_{i}p_{j}.$$

This analysis was conducted using R “genetics” v 1.3.8 packages (https://cran.r-project.org/web/packages/genetics/index.html) and visualized using the R package of “LDheatmap” [57].

### Deposited data

All of the primary sequencing data (including Illumina and PacBio DNA sequencing and CENP-A ChIP-seq), as well as genome assemblies, have been deposited under NCBI BioProject Accession no. PRJNA200571 and EBI study accession no. PRJEB19939.

## Supporting information

S1 Fig Validation of the *C*. *amylolentus* genome assemblies by Southern hybridization. On each row at the far left is a gel image of the CHEF electrophoresis separation of chromosomes in the *2 C*. *amylolentus* isolates (CBS6039 and CBS6273), with *Saccharomyces cerevisiae* chromosomes serving as size markers. The figure at the bottom right corner illustrates the distribution of the probes in the CBS6039 genome. Probes on chromosomes 10 and 11 were not included as the assemblies of these 2 chromosomes are supported by the linkage map analyses in which markers from each chromosome clustered together and formed a single linkage group, and by previous published chromoblot analysis [22].
(TIF)

S2 Fig Synteny analyses of the *MAT* loci in *C*. *amylolentus* and *C*. *neoformans* species. The *MAT* loci (*HD* and *P/R*) from the *2 C*. *amylolentus* (C.a.) isolates (CBS6039 and CBS6273) are compared to the *MAT* alleles (**a** and α) from species within the human pathogenic *Cryptococcus* species complex: *C*. *neoformans* (*C*. *neo*.) and *C*. *deneoformans* (*C*. *den*.). Red color highlights the genes that define the *HD* locus (*SXI1* and *SXI2*) and *P/R* locus (mating pheromones and *STE3*); blue color highlights the genes that are present within the *MAT* locus in the pathogenic *Cryptococcus* species complex; gray color highlights the genes that are present within the *C*. *amylolentus MAT* loci but are absent from the *MAT* locus in *C*. *neoformans* and *C*. *deneoformans*.
(TIF)

S3 Fig Identification and characterization of centromeric regions in the CBS6039 genome. (A) Illustration of read depth of the mCherry-Cse4 ChIP-seq data along each of the 14 chromosomes in the CBS6039 genome. (B) The upper panel is an illustration of read depth along chromosome 7 when the mCherry-Cse4 ChIP-seq data was mapped onto the CBS6039 genome. As shown in the magnified section, the region that had the highest ChIP-seq read coverage (zoomed in section, top panel) also showed low levels of transcriptional activity based on RNA-seq analysis (zoomed in section, middle panel, where blue bars indicate RNA-seq read depth). Additionally, bioinformatic analyses also showed that ORFs are sparsely distributed in these regions, and the majority of these ORFs are transposable element related (zoomed in section, bottom panel, where the rectangular box in the middle indicates the centromeric region of chromosome 7, with blocks of blue, orange, and green colors within depicting the different types of transposable elements, and the 2 block arrows illustrating the flanking ORFs).
(TIF)

S4 Fig Genome comparison between *C*. *amylolentus* strain CBS6039 and chromosome 5 of *C*. *neoformans* strain H99. Shown here are distributions of BLAST hits in the CBS6039 genome, using nucleotide sequence of chromosome 5 in the H99 genome, on which the *MAT* locus is located. (A) Illustration that includes all 14 chromosomes in the CBS6039 genome. (B) Illustration that only includes the 5 chromosomes in the CBS6039 genome from which H99 chromosome 5 has significant hits. Abbreviations: CEN, centromere flanking region; *MAT*, *C*. *neoformans MAT* locus; *P/R*, *C*. *amylolentus P/R MAT* locus; *HD*, *C*. *amylolentus HD MAT* locus.
(TIF)

S5 Fig The SNP distribution in the genome in meiotic progeny from a cross between strains CBS6039 and CBS6273. The blue color indicates SNPs that correspond to the genomic sequence of strain CBS6039, and the red color indicates SNPs that correspond to the genomic sequence of strain CBS6273. Meiotic progeny from 2 individual basidia (#1 and #2), as well as 2 random basidiospores, were analyzed. For basidium #2, 2 additional basidiospores, #4 and #6, that are genetically identical to basidiospores #3 and #5, respectively, were also included.
(TIF)

S6 Fig Heat maps of linkage disequilibrium (LD) for chromosomes 10 and 11. The LD was estimated based on the r^2^ statistic. The numbers along the top and left sides of each diagram indicate genetic markers on chromosomes 10 and 11, respectively (see S5 Table for detailed information on the markers). The color of the squares in the matrix indicates the r^2^ value between the 2 markers, and the darker the square, the higher the r^2^ value, and consequently, the stronger the linkage between the 2 markers. Green blocks highlight markers that are located within the *P/R* and *HD* loci, and red blocks highlight markers that are flanking or located within the centromeres. The diagonal brackets highlight blocks with high LD scores that are present on chromosomes 10 and 11. Data used to generate the figure can be found at NCBI BioProject with accession no. PRJNA200571 and at EBI with study accession no. PRJEB19939.
(TIF)

S1 Table Insertions/deletions and chromosomal rearrangements identified between the genomes of CBS6039 and CBS6273. (PDF)

S2 Table Pathways enriched in the genomes of *C*. *amylolentus* compared to those of pathogenic *Cryptococcus* species. (PDF)

S3 Table List of ORFs identified that are either flanking or located within the candidate centromeric regions in the CBS6039 genome. (PDF)

S4 Table Summary of the meiotic progeny analyzed in this study. (PDF)

S5 Table Markers and primers used in this study. (PDF)

## Abbreviations

CEN: centromere
CENP-A: centromere binding protein Cse4
CHEF: contour-clamped homogeneous electric field
ChIP-seq: chromatin immunoprecipitation sequencing
LD: linkage disequilibrium
LTR: Long Terminal Repeat
MAT: mating type locus
NPD: non-parental ditype
PD: parental ditype
RNA-seq: RNA sequencing
Tcen: transposons in centromeres
TT: tetratype
