# Supplementary figures and images for: Fungal genome and mating system transitions facilitated by chromosomal translocations involving intercentromeric recombination
Source: PLoS Biol. 2017 Aug 11;15(8):e2002527. doi: 10.1371/journal.pbio.2002527 (PMC5568439; doi:10.1371/journal.pbio.2002527)

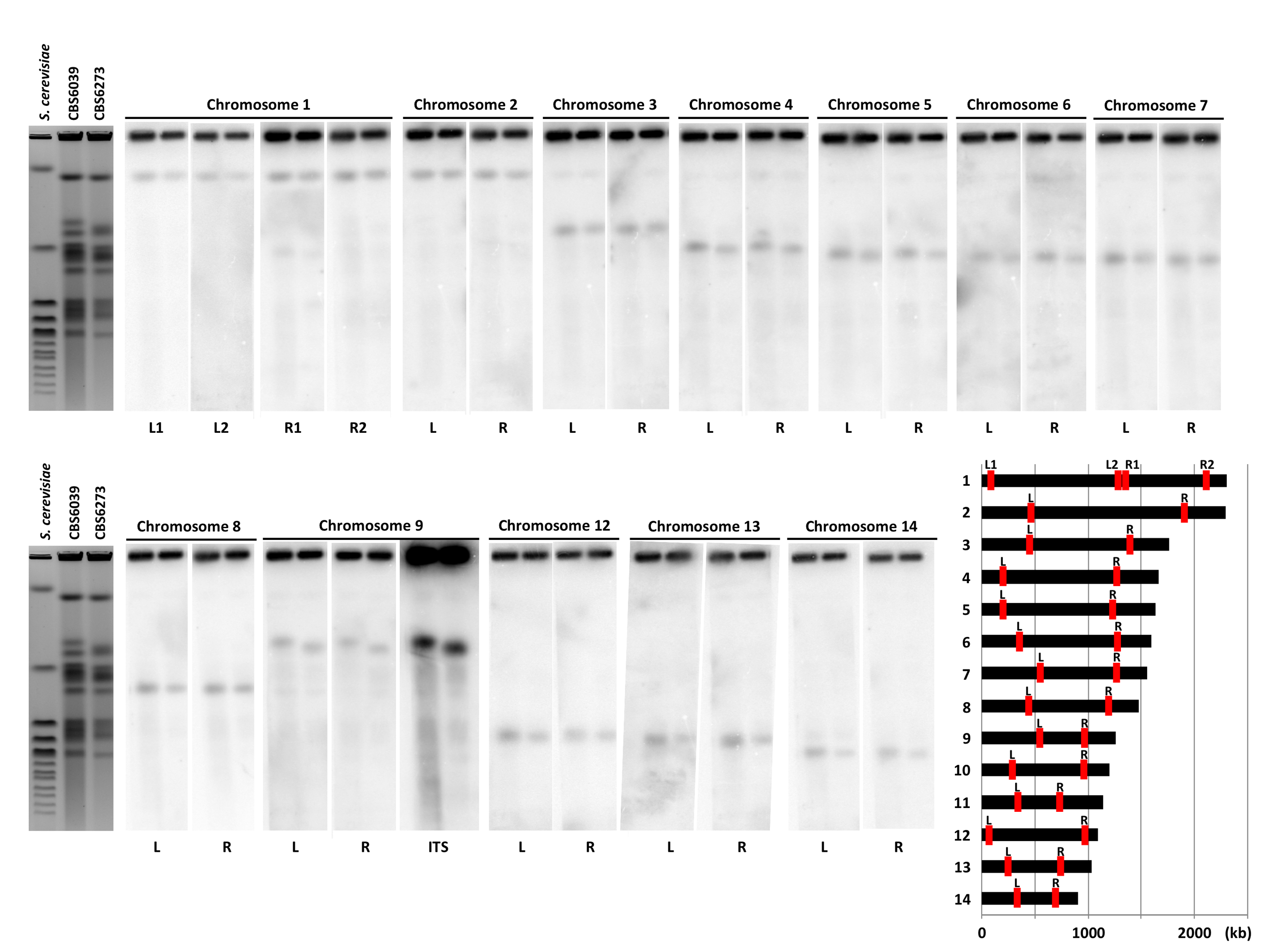

Supplement: S1 Fig — On each row at the far left is a gel image of the CHEF electrophoresis separation of chromosomes in the 2 C. amylolentus isolates (CBS6039 and CBS6273), with Saccharomyces cerevisiae chromosomes serving as size markers. The figure at the bottom right corner illustrates the distribution of the probes in the CBS6039 genome. Probes on chromosomes 10 and 11 were not included as the assemblies of these 2 chromosomes are supported by the linkage map analyses in which markers from each chromosome clustered together and formed a single linkage group, and by previous published chromoblot analysis [22]. (TIF) [file pbio.2002527.s001.tif]

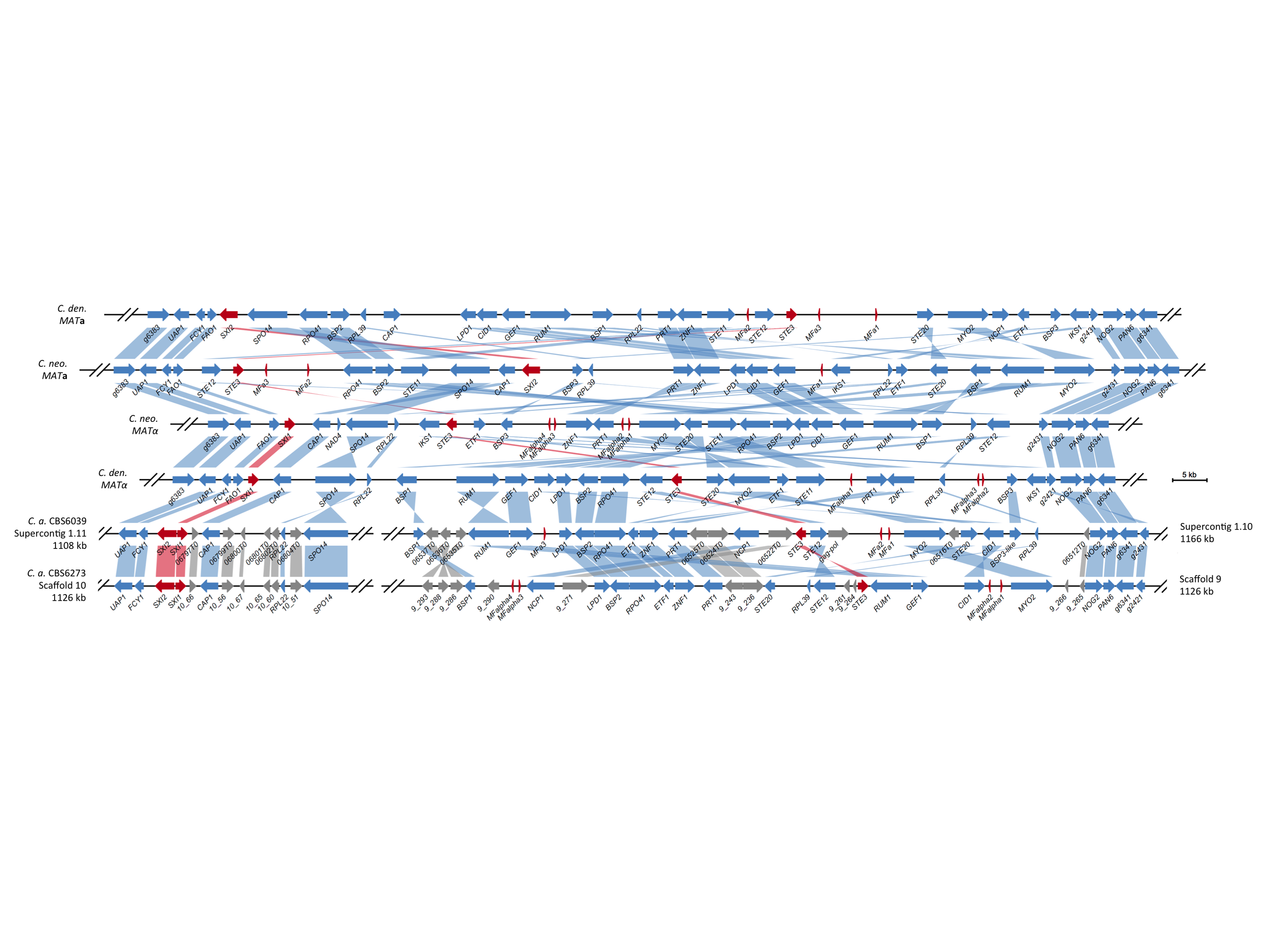

Supplement: S2 Fig — The MAT loci (HD and P/R) from the 2 C. amylolentus (C.a.) isolates (CBS6039 and CBS6273) are compared to the MAT alleles (a and α) from species within the human pathogenic Cryptococcus species complex: C. neoformans (C. neo.) and C. deneoformans (C. den.). Red color highlights the genes that define the HD locus (SXI1 and SXI2) and P/R locus (mating pheromones and STE3); blue color highlights the genes that are present within the MAT locus in the pathogenic Cryptococcus species complex; gray color highlights the genes that are present within the C. amylolentus MAT loci but are absent from the MAT locus in C. neoformans and C. deneoformans. (TIF) [file pbio.2002527.s002.tif]

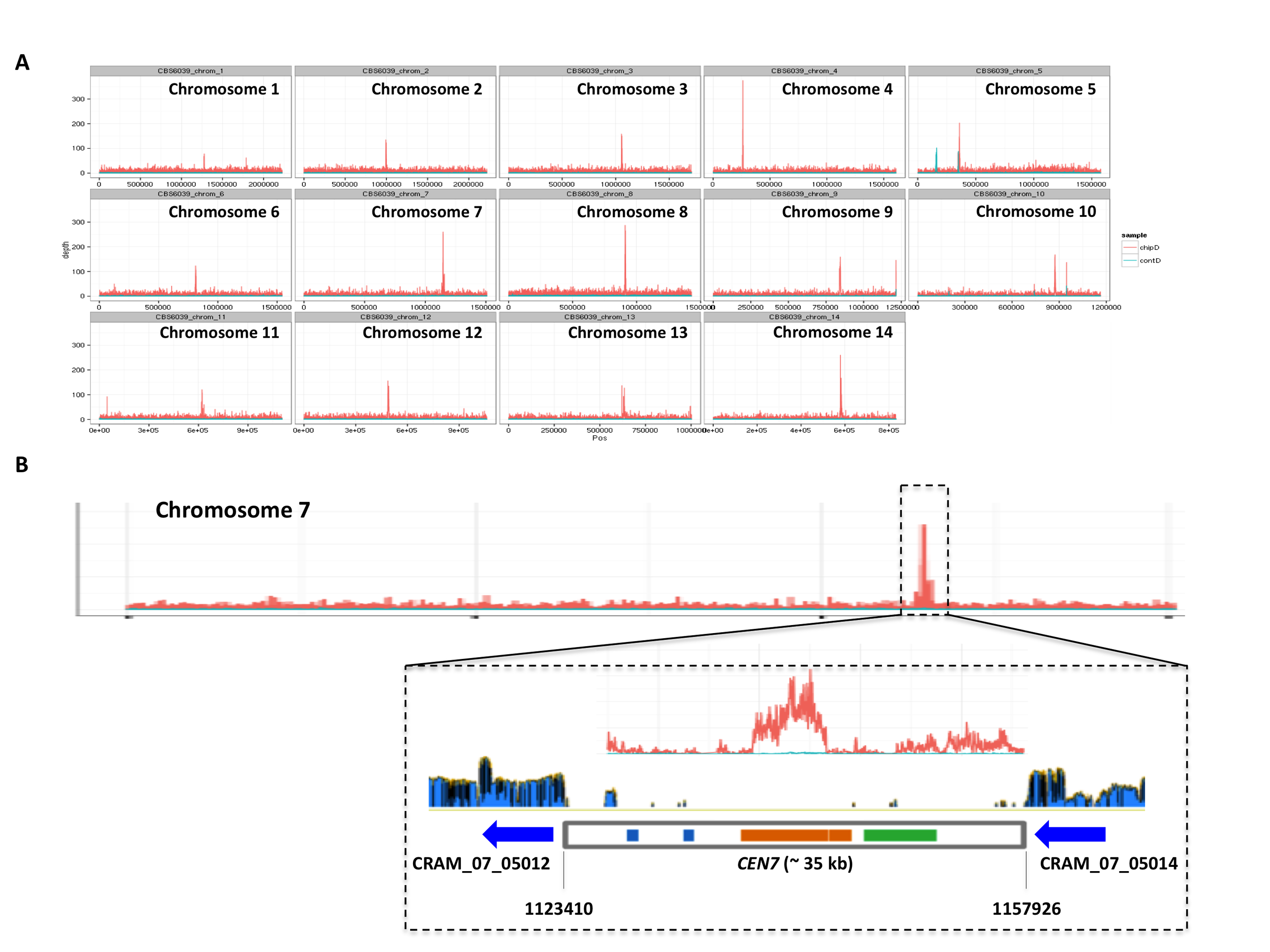

Supplement: S3 Fig — (A) Illustration of read depth of the mCherry-Cse4 ChIP-seq data along each of the 14 chromosomes in the CBS6039 genome. (B) The upper panel is an illustration of read depth along chromosome 7 when the mCherry-Cse4 ChIP-seq data was mapped onto the CBS6039 genome. As shown in the magnified section, the region that had the highest ChIP-seq read coverage (zoomed in section, top panel) also showed low levels of transcriptional activity based on RNA-seq analysis (zoomed in section, middle panel, where blue bars indicate RNA-seq read depth). Additionally, bioinformatic analyses also showed that ORFs are sparsely distributed in these regions, and the majority of these ORFs are transposable element related (zoomed in section, bottom panel, where the rectangular box in the middle indicates the centromeric region of chromosome 7, with blocks of blue, orange, and green colors within depicting the different types of transposable elements, and the 2 block arrows illustrating the flanking ORFs). (TIF) [file pbio.2002527.s003.tif]

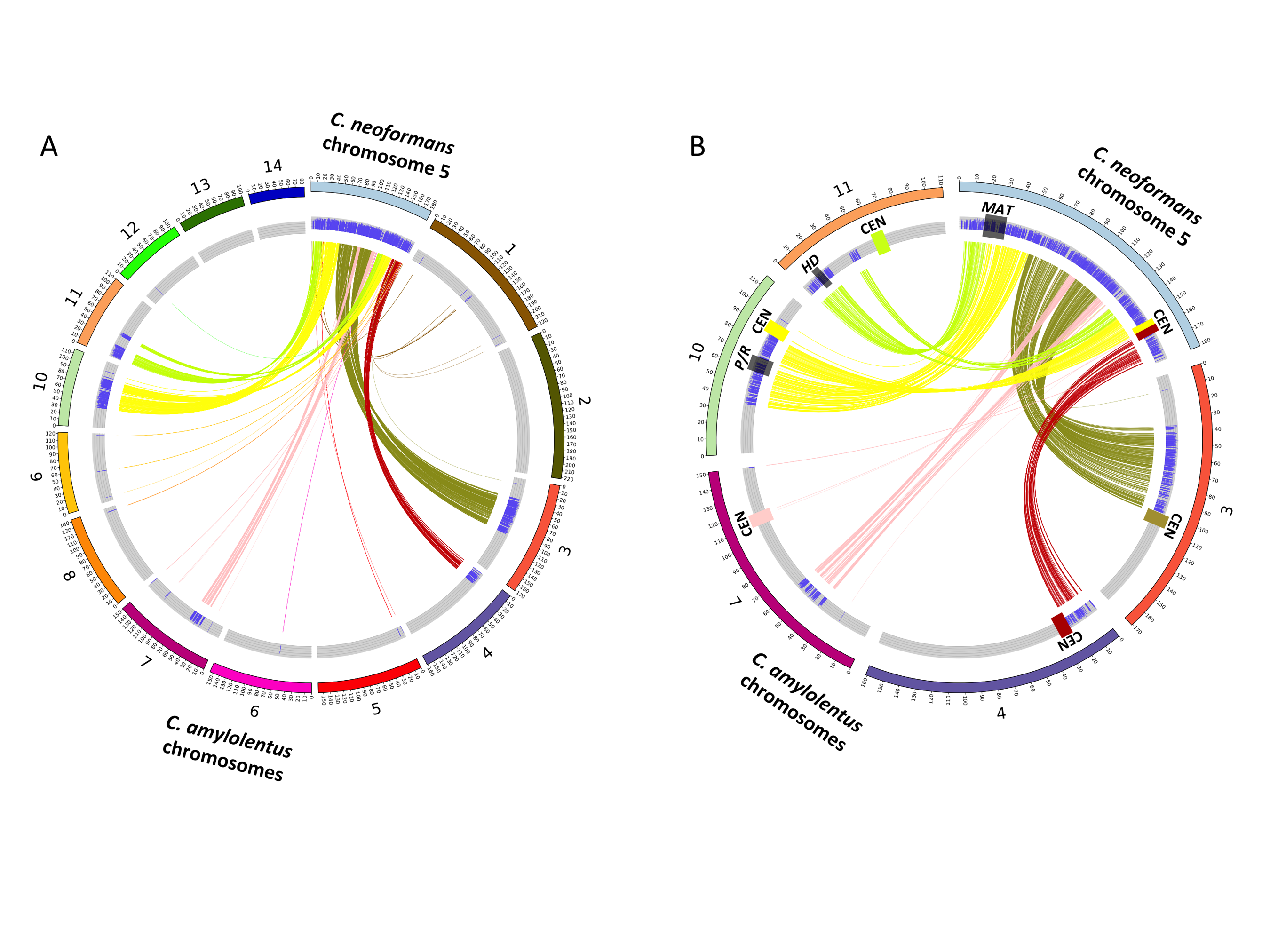

Supplement: S4 Fig — Shown here are distributions of BLAST hits in the CBS6039 genome, using nucleotide sequence of chromosome 5 in the H99 genome, on which the MAT locus is located. (A) Illustration that includes all 14 chromosomes in the CBS6039 genome. (B) Illustration that only includes the 5 chromosomes in the CBS6039 genome from which H99 chromosome 5 has significant hits. Abbreviations: CEN, centromere flanking region; MAT, C. neoformans MAT locus; P/R, C. amylolentus P/R MAT locus; HD, C. amylolentus HD MAT locus. (TIF) [file pbio.2002527.s004.tif]

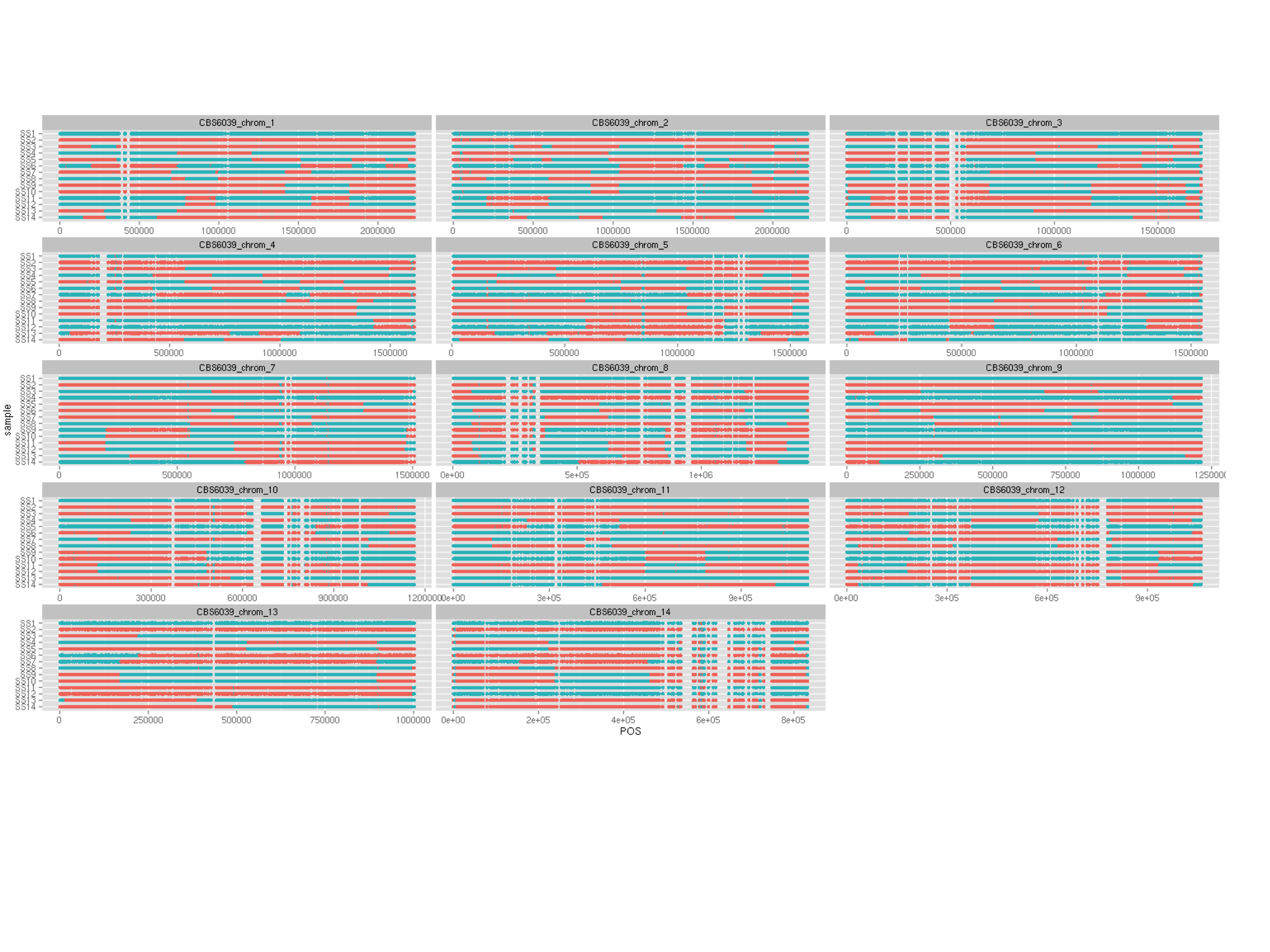

Supplement: S5 Fig — The blue color indicates SNPs that correspond to the genomic sequence of strain CBS6039, and the red color indicates SNPs that correspond to the genomic sequence of strain CBS6273. Meiotic progeny from 2 individual basidia (#1 and #2), as well as 2 random basidiospores, were analyzed. For basidium #2, 2 additional basidiospores, #4 and #6, that are genetically identical to basidiospores #3 and #5, respectively, were also included. (TIF) [file pbio.2002527.s005.tif]

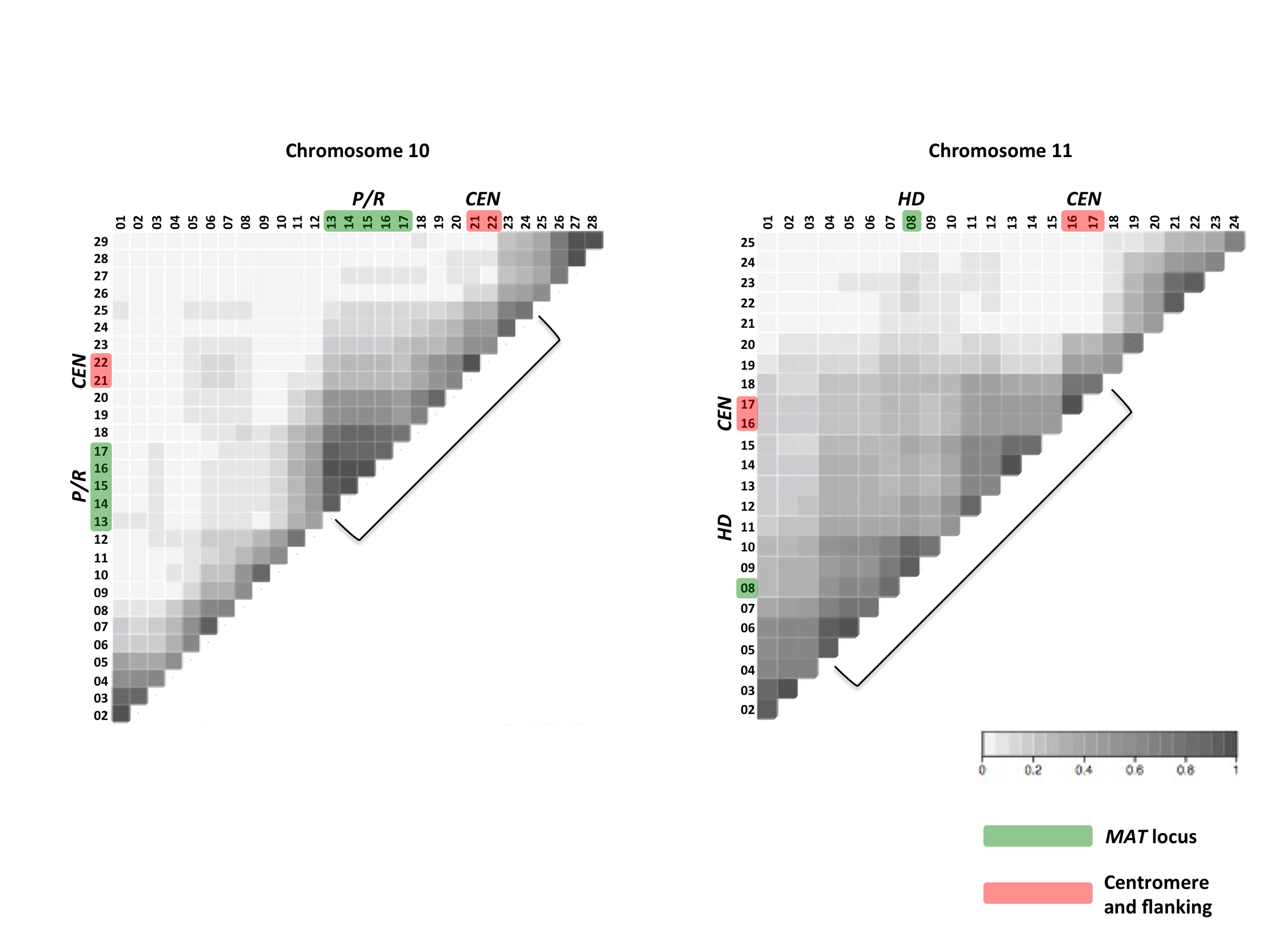

Supplement: S6 Fig — The LD was estimated based on the r2 statistic. The numbers along the top and left sides of each diagram indicate genetic markers on chromosomes 10 and 11, respectively (see S5 Table for detailed information on the markers). The color of the squares in the matrix indicates the r2 value between the 2 markers, and the darker the square, the higher the r2 value, and consequently, the stronger the linkage between the 2 markers. Green blocks highlight markers that are located within the P/R and HD loci, and red blocks highlight markers that are flanking or located within the centromeres. The diagonal brackets highlight blocks with high LD scores that are present on chromosomes 10 and 11. Data used to generate the figure can be found at NCBI BioProject with accession no. PRJNA200571 and at EBI with study accession no. PRJEB19939. (TIF) [file pbio.2002527.s006.tif]
