# Supplementary material for: Fungal genome and mating system transitions facilitated by chromosomal translocations involving intercentromeric recombination
Source: PLoS Biol. 2017 Aug 11;15(8):e2002527. doi: 10.1371/journal.pbio.2002527 (PMC5568439; doi:10.1371/journal.pbio.2002527)
Supplement: S1 Table — (PDF) [file pbio.2002527.s007.pdf]

Table S1. Insertion/deletion and chromosomal rearrangements identified between the genomes of CBS6039 and CBS6273.

Type of event	CBS6039 (chromosome)	CBS6273 (chromosome)	Size of event (CBS6039)	Size of event (CBS6273)	Midpoint of event (CBS6039)	Midpoint of event (CBS6273)
duplication in CBS6273	7	7	-4127	5843	101308	38857
insertion/duplication in CBS6273	2	1	-1835	100	246303	232073
duplication in both strains	2	1	-738	-738	20279	20673
duplication in both strains	2	1	-738	-738	22633	22290
insertion/duplication in CBS6273	1	2	-651	670	2112006	113742
insertion/duplication in CBS6273	7	7	-536	929	730799	665745
insertion/duplication in CBS6273	9	9	-449	271	248661	249865
inversion in CBS6273	9	9	-449	2421	275402	277190
duplication in both strains	1	2	-387	-1455	2105068	120390
insertion/duplication in CBS6273	11	11	-334	1432	796173	815415
insertion/duplication in CBS6273	4	4	-333	287	1408547	1399651
insertion/duplication in CBS6273	10	10	-273	1764	100758	108187
insertion/duplication in CBS6273	14	14	-269	4822	767573	770942
insertion/duplication in CBS6273	13	13	-248	0	813203	814907
insertion/duplication in CBS6273	14	14	-198	88	720579	721099
insertion/duplication in CBS6273	10	10	-165	39	60666	66392
duplication in CBS6273	6	6	-158	0	1212411	329437
insertion/duplication in CBS6273	2	1	-107	3017	1876313	1885731
insertion/duplication in CBS6273	14	14	-98	116	42585	49916
insertion/duplication in CBS6273	13	13	-94	256	86707	89156
insertion/duplication in CBS6273	10	10	-75	151	1076988	1089725
insertion/duplication in CBS6273	1	2	-69	4	951404	1272971
insertion/duplication in CBS6273	9	9	-61	100	305899	307092
duplication in both strains	6	6	-49	-49	122023	1406730
duplication in both strains	6	6	-49	-49	122214	1406539
insertion/duplication in CBS6273	8	8	-45	18	157072	160854
insertion/duplication in CBS6273	13	13	-44	1	947630	949545
insertion/duplication in CBS6273	14	14	-43	135	593511	593803
insertion/duplication in CBS6273	3	3	-40	108	99169	1607641
insertion/duplication in CBS6273	5	5	-39	252	30160	31158
insertion/duplication in CBS6273	11	11	-35	147	533340	533866
insertion/duplication in CBS6273	13	13	-28	100	437040	437300
insertion/duplication in CBS6273	10	10	-22	537	112219	120971

Type of event	CBS6039 (chromosome)	CBS6273 (chromosome)	Size of event (CBS6039)	Size of event (CBS6273)	Midpoint of event (CBS6039)	Midpoint of event (CBS6273)
insertion/duplication in CBS6273	6	6	-22	346	194521	1334403
insertion/duplication in CBS6273	8	8	-14	37	324290	327814
inversion in CBS6273	14	14	-13	NA	194906	793477
insertion/duplication in CBS6273	12	12	-13	195	812917	254729
insertion/duplication in CBS6039	10	10	-12	57	226213	232611
insertion/duplication in CBS6273	4	4	-12	44	1403508	1394261
duplication in both strains	2	1	-12	-3183	2217497	2221178
insertion/duplication in CBS6273	13	13	-8	264	974605	977194
insertion/duplication in CBS6273	6	6	-7	194	15402	1513821
insertion/duplication in CBS6273	12	12	-7	305	50665	1043019
insertion/duplication in CBS6273	12	12	-7	5231	899318	165581
insertion/duplication in CBS6273	5	5	-7	37	1337604	1324437
insertion/duplication in CBS6273	13	13	-6	530	86650	88656
insertion/duplication in CBS6273	5	5	-6	309	151896	153489
insertion/duplication in CBS6273	4	4	-6	309	966651	968297
insertion/duplication in CBS6273	2	1	-6	1020	1329821	1337210
insertion/duplication in CBS6273	4	4	-5	312	11124	11188
insertion/duplication in CBS6273	7	scaffold00016	-5	5101	20306	23174
insertion/duplication in CBS6273	2	1	-5	310	44773	44841
insertion/duplication in CBS6273	4	4	-5	301	54781	56038
insertion/duplication in CBS6273	10	10	-5	310	90301	96288
insertion/duplication in CBS6273	10	10	-5	383	96082	102375
insertion/duplication in CBS6273	14	14	-5	299	176368	184286
insertion/duplication in CBS6273	10	10	-5	194	1092214	1105200
insertion/duplication in CBS6273	10	10	-5	267	1136907	1146660
insertion/duplication in CBS6273	2	1	-5	2434	1212243	1217874
insertion/duplication in CBS6273	3	3	-5	531	1351691	355659
insertion/duplication in CBS6273	4	4	-5	300	1369141	1358861
insertion/duplication in CBS6273	4	4	-5	429	1425755	1417384
insertion/duplication in CBS6273	5	5	-5	193	1491172	1475606
insertion/duplication in CBS6273	1	2	-5	1062	2070602	155529
insertion/duplication in CBS6273	1	2	-3	859	525121	1699593
insertion/duplication in CBS6273	10	10	-3	198	1093596	1106771
insertion/duplication in CBS6273	7	7	-2	64	779154	714849

Type of event	CBS6039 (chromosome)	CBS6273 (chromosome)	Size of event (CBS6039)	Size of event (CBS6273)	Midpoint of event (CBS6039)	Midpoint of event (CBS6273)
insertion/duplication in CBS6273	12	12	-2	190	901874	160322
duplication in CBS6039	5	5	-2	-2700	1441641	1427278
insertion/duplication in CBS6039	2	1	-2	-1890	2148629	2158743
insertion/duplication in CBS6273	10	10	-1	5936	43804	46462
insertion/duplication in CBS6273	7	7	-1	1252	117544	57780
insertion/duplication in CBS6273	12	12	-1	411	918101	143787
insertion/duplication in CBS6273	6	6	-1	167	1364806	177067
deletion in CBS6039	3	3	0	50	2301	1704827
duplication in CBS6039	4	4	0	-67	27055	27240
deletion in CBS6039	6	6	0	237	39037	1489959
deletion in CBS6039	5	5	0	2740	50704	52874
deletion in CBS6039	8	8	0	2539	54762	56997
deletion in CBS6039	14	14	0	317	60650	68235
deletion in CBS6039	5	5	0	94	116542	119497
deletion in CBS6039	1	2	0	95	119004	2105730
deletion in CBS6039	10	10	0	350	161877	168082
deletion in CBS6039	12	12	0	547	168484	924795
deletion in CBS6039	7	7	0	100	230296	164493
deletion in CBS6039	10	10	0	2126	307356	314871
deletion in CBS6039	11	11	0	13	334352	334816
deletion in CBS6039	1	2	0	105	335651	1889585
deletion in CBS6039	6	6	0	36	433780	1095345
insertion in CBS6039	11	11	0	-348	581170	594849
duplication in CBS6039	8	8	0	-319	675505	678945
deletion in CBS6039	8	8	0	186	680764	684138
deletion in CBS6039	14	14	0	85	786642	786626
deletion in CBS6039	10	10	0	143	871171	884416
deletion in CBS6039	9	9	0	416	892573	897268
deletion in CBS6039	13	13	0	811	955114	957441
deletion in CBS6039	4	4	0	125	1001557	1003415
deletion in CBS6039	10	10	0	12	1014486	1027148
duplication in CBS6039	8	8	0	-119	1039646	1043670
deletion in CBS6039	3	3	0	52	1062023	645655
deletion in CBS6039	6	6	0	145	1239025	302482

Type of event	CBS6039 (chromosome)	CBS6273 (chromosome)	Size of event (CBS6039)	Size of event (CBS6273)	Midpoint of event (CBS6039)	Midpoint of event (CBS6273)
deletion in CBS6039	4	4	0	4802	1481222	1473769
duplication in CBS6039	3	3	0	-680	1567121	140397
inversion in CBS6273	5	5	0	2	1575044	1559622
deletion in CBS6039	3	3	0	35	1588509	119331
inversion in CBS6273	4	4	0	167	1608362	1599962
deletion in CBS6039	1	2	0	776	1854593	370952
deletion in CBS6039	9	9	1	209	121912	121986
deletion in CBS6039	12	12	1	2369	508691	561252
deletion in CBS6039	4	4	1	631	1369479	1359664
insertion/duplication in CBS6039	9	9	2	-37	1164667	1160572
deletion in CBS6039	1	2	2	132	1196129	1028382
deletion in CBS6039	3	3	3	108	59504	1647433
deletion in CBS6039	10	10	3	118	400584	409219
insertion/duplication in CBS6039	5	5	3	-49	1548781	1533366
insertion/duplication in CBS6039	13	13	4	-275	35056	36806
deletion in CBS6039	13	13	4	151	51583	53262
insertion/duplication in CBS6039	4	4	4	-32	80529	81475
deletion in CBS6039	6	6	4	165	116702	1412148
deletion in CBS6039	4	4	4	250	1370447	1361082
insertion/duplication in CBS6039	3	3	5	-218	183877	1523119
deletion in CBS6039	7	7	5	122	1372282	1315371
deletion in CBS6039	3	3	6	268	1529070	178014
insertion/duplication in CBS6039	8	8	9	-209	787682	791043
deletion in CBS6039	13	13	10	1087	1132	2487
deletion in CBS6039	8	8	10	535	52294	52997
deletion in CBS6039	9	9	10	100	1075209	1078971
insertion in CBS6039	6	6	13	0	205096	1323896
deletion in CBS6039	11	11	15	150	98772	98266
insertion/duplication in CBS6039	2	1	15	-132	671293	657962
deletion in CBS6039	12	12	15	322	1039872	22068
deletion in CBS6039	6	6	15	107	1429306	112419
deletion in CBS6039	11	11	16	453	50402	51087
insertion in CBS6039	3	3	17	0	1536211	170741
insertion in CBS6039	6	6	18	0	201337	1327640

Type of event	CBS6039 (chromosome)	CBS6273 (chromosome)	Size of event (CBS6039)	Size of event (CBS6273)	Midpoint of event (CBS6039)	Midpoint of event (CBS6273)
insertion/duplication in CBS6039	10	10	18	-21	1067543	1080188
insertion in CBS6039	5	5	18	0	1114534	1101327
insertion in CBS6039	6	6	20	0	223747	1305252
deletion in CBS6039	6	6	21	304	1173120	368498
deletion in CBS6039	11	11	27	191	795299	813573
insertion/duplication in CBS6039	1	2	34	-4	2073272	152344
insertion/duplication in CBS6039	7	7	36	-54	392518	326755
inversion in CBS6273	13	13	37	1942	582	2488
deletion in CBS6039	6	6	37	239	1354293	187768
insertion in CBS6039	3	3	39	0	849424	857844
insertion/duplication in CBS6039	3	3	40	-2	142986	1563898
insertion in CBS6039	3	3	42	0	1536574	170409
insertion in CBS6039	6	6	43	0	314907	1214143
insertion/duplication in CBS6039	12	12	43	-2	391203	702437
insertion in CBS6039	1	2	45	0	254193	1971096
insertion in CBS6039	2	1	45	0	1585910	1593773
insertion in CBS6039	11	11	48	0	1047845	1066228
insertion in CBS6039	1	2	56	2	589084	1635224
insertion/duplication in CBS6039	6	6	65	-3	432988	1096121
insertion/duplication in CBS6039	3	3	77	-172	1518913	188183
insertion in CBS6039	6	6	84	0	1238243	303298
insertion/duplication in CBS6039	7	scaffold00016	88	-6	46436	51776
insertion in CBS6039	6	6	96	0	1226341	315150
insertion in CBS6039	11	11	102	0	466447	466910
insertion in CBS6039	3	3	104	1	725398	981818
insertion in CBS6039	12	12	105	1	1038125	23916
deletion in CBS6039	10	10	105	904	1048312	1060603
insertion/duplication in CBS6039	2	1	107	-18	140786	126371
insertion/duplication in CBS6039	1	2	108	-90	1660211	565541
insertion in CBS6039	2	1	116	1	43120	43087
deletion in CBS6039	8	8	117	675	3260	3460
insertion in CBS6039	7	7	124	0	1096446	1032073
deletion in CBS6039	4	4	124	991	1467642	1459956
insertion in CBS6039	1	2	131	2	54215	2170491

Type of event	CBS6039 (chromosome)	CBS6273 (chromosome)	Size of event (CBS6039)	Size of event (CBS6273)	Midpoint of event (CBS6039)	Midpoint of event (CBS6273)
insertion in CBS6039	5	5	140	0	852178	839079
inversion in CBS6273	14	14	141	2	829659	842582
insertion in CBS6039	5	5	146	1	125981	127515
insertion/duplication in CBS6039	6	6	146	-22	1525550	16613
insertion in CBS6039	7	7	147	3	128019	68982
insertion/duplication in CBS6039	6	6	150	-23	100024	1428882
insertion/duplication in CBS6039	11	11	152	-1	66832	67682
insertion/duplication in CBS6039	6	6	152	-186	1489605	52330
inversion in CBS6273	6	6	154	0	1211762	329762
deletion in CBS6039	11	11	165	951	28607	28649
insertion in CBS6039	10	10	165	0	1100936	1113992
insertion/duplication in CBS6039	8	8	167	-1	1277335	1280168
insertion in CBS6039	5	5	169	0	237830	226250
insertion in CBS6039	9	9	169	0	740441	741598
insertion in CBS6039	8	8	184	0	1392504	1389977
insertion/duplication in CBS6039	2	1	189	-4	686051	672538
insertion in CBS6039	13	13	191	1	528365	528611
insertion/duplication in CBS6039	14	14	195	-6	794265	807906
insertion/duplication in CBS6039	4	4	197	-5	75941	77002
insertion/duplication in CBS6039	13	13	200	-8	99509	101756
insertion in CBS6039	3	3	202	28	1558126	148967
insertion/duplication in CBS6039	4	4	216	-6	1366027	1356020
insertion/duplication in CBS6039	1	2	216	-6	2205236	22044
inversion in CBS6273	1	2	220	12	2225656	1843
insertion in CBS6039	6	6	221	6	200648	1328212
inversion in CBS6273	11	11	224	2	1106840	1125333
insertion/duplication in CBS6039	11	11	226	-6	1084401	1102647
insertion/duplication in CBS6039	12	12	247	-5	276823	816663
insertion/duplication in CBS6039	5	5	249	-1	76538	79664
insertion/duplication in CBS6039	13	13	265	-1	967458	970044
insertion/duplication in CBS6039	1	2	267	-5	2167995	57546
insertion/duplication in CBS6039	1	2	304	-11	2180102	45734
insertion/duplication in CBS6039	4	4	310	-5	1367891	1357615
insertion/duplication in CBS6039	12	12	311	-5	18114	1075569

Type of event	CBS6039 (chromosome)	CBS6273 (chromosome)	Size of event (CBS6039)	Size of event (CBS6273)	Midpoint of event (CBS6039)	Midpoint of event (CBS6273)
insertion/duplication in CBS6039	5	5	315	-23	75377	78797
insertion/duplication in CBS6039	10	10	347	-294	944534	957529
insertion/duplication in CBS6039	12	12	354	-3	186269	906916
insertion in CBS6039	7	7	374	2	118211	58887
insertion in CBS6039	1	2	384	0	1152712	1071691
insertion in CBS6039	1	2	384	76	1306576	918981
inversion in CBS6273	8	8	401	0	1427230	1424392
deletion in CBS6039	12	12	428	2452	617923	449769
insertion/duplication in CBS6039	11	11	459	-255	793599	812159
insertion/duplication in CBS6039	10	10	512	-4	1042369	1054502
insertion/duplication in CBS6039	6	6	533	-192	1242164	299630
insertion in CBS6039	1	2	593	22	186585	2038368
insertion/duplication in CBS6039	8	8	620	-6	1298445	1300895
insertion/duplication in CBS6039	1	2	724	-508	2184885	41715
insertion/duplication in CBS6039	14	14	750	-1	281811	282055
insertion/duplication in CBS6039	5	5	775	-590	692676	680349
deletion in CBS6039	11	11	779	13820	560302	567636
deletion in CBS6039	7	7	838	4457	1432721	1378297
insertion in CBS6039	2	1	995	107	219232	204493
insertion/duplication in CBS6039	9	9	1007	-1	1131700	1130522
insertion in CBS6039	11	11	1029	127	712924	732304
insertion/duplication in CBS6039	2	1	1203	-54	2187555	2191876
insertion/duplication in CBS6039	11	11	1344	-2	93263	93363
insertion/duplication in CBS6039	5	5	1486	-4	124972	127325
insertion/duplication in CBS6039	1	2	1500	-86	2055678	170194
insertion/duplication in CBS6039	11	11	1704	-11	973647	992929
insertion/duplication in CBS6039	13	13	1983	-6	158962	160107
insertion/duplication in CBS6039	14	14	1984	-5	797421	809966
insertion/duplication in CBS6039	10	10	2248	-3	117573	125506
insertion/duplication in CBS6039	7	7	2335	-1	145615	85138
insertion in CBS6039	9	9	2403	74	1151893	1149033
inversion in CBS6273	9	9	3126	-170	290278	292953
insertion/duplication in CBS6039	4	4	3139	-2	1489229	1482549
insertion/duplication in CBS6039	2	1	3300	-945	2158732	2165808

Type of event	CBS6039 (chromosome)	CBS6273 (chromosome)	Size of event (CBS6039)	Size of event (CBS6273)	Midpoint of event (CBS6039)	Midpoint of event (CBS6273)
insertion/duplication in CBS6039	7	7	3385	-743	188011	124204
insertion/duplication in CBS6039	10	10	3387	-233	1103229	1114379
inversion in CBS6273	9	9	4275	172	1213248	1201367
insertion/duplication in CBS6039	8	8	4664	-58	1320415	1320309
insertion in CBS6039	9	9	5428	765	1103900	1105555
insertion in CBS6039	4	4	11819	6	1106865	1102898
insertion/duplication in CBS6039	2	1	11919	-2628	55384	48349
insertion in CBS6039	5	5	13155	14	159052	154234
