# Supplementary material for: Fungal genome and mating system transitions facilitated by chromosomal translocations involving intercentromeric recombination
Source: PLoS Biol. 2017 Aug 11;15(8):e2002527. doi: 10.1371/journal.pbio.2002527 (PMC5568439; doi:10.1371/journal.pbio.2002527)
Supplement: S2 Table — (PDF) [file pbio.2002527.s008.pdf]

Table S2. Pathways enriched in the genomes of *C. amylolentus* compared to those of pathogenic *Cryptococcus* species

Pathway	CRAM_CBS6039 [1]	CRAM_CBS6273 [1]	Pathogenic_H99 [2]	Pathogenic_JEC21 [2]	Pathogenic_R265 [2]	Pathogenic_WM276 [2]	log2(CRAM/ Pathogenic)	rawPvalue	Qvalue
PF13460.1|NAD_binding_10 [NADH(P)-binding]	56	54	20	23	14	16	2.43	3.20E-11	1.33E-07
PF00201.13|UDPGT [UDP-glucoronosyl_and_UDP-glucosyl_transferase]	14	14	2	2	1	2	3.78	1.12E-07	2.34E-04
PF04616.9|Glyco_hydro_43 [Glycosyl_hydrolases_family_43]	5	5	0	0	0	0	6.51	3.47E-05	3.24E-02
PF05368.8|NmrA [NmrA-like_family]	30	29	12	16	9	10	2.16	3.90E-05	3.24E-02

[1]: "CRAM" indicates *C. amylolentus*.
[2]: "Pathogenic" indicates species within the human pathogenic *Cryptococcus* species complex.
