# Supplementary material for: Fungal genome and mating system transitions facilitated by chromosomal translocations involving intercentromeric recombination
Source: PLoS Biol. 2017 Aug 11;15(8):e2002527. doi: 10.1371/journal.pbio.2002527 (PMC5568439; doi:10.1371/journal.pbio.2002527)
Supplement: S3 Table — (PDF) [file pbio.2002527.s009.pdf]

Table S3. List of ORFs identified that are flanking or within the candidate centromeric regions in CBS6039

Chromosome	Region	Coordinates (size; bp)	ORF (size; no. of amino acids) [1]	Description [2]	Ortholog in the H99 genome [3]
1	Flanking (left)	1259704-1261928	CRAM_01_00527		**CNAG_01780**
	Intra-centromere	1261929-1292020 (30,091)	CRAM_01_00528 (125)	Transposon	
			CRAM_01_00530 (108)	Transposon	
	Flanking (right)	1292021-1293761	CRAM_01_00532		**CNAG_03796**
2	Flanking (left)	979876-981299	CRAM_02_01330		**CNAG_01788**
	Intra-centromere	981300-1003798 (22,498)	CRAM_02_01331 (266)	Transposon	
	Flanking (right)	1003799-1005713	CRAM_02_01332		**CNAG_03805**
3	Flanking (left)	1036140-1037292	CRAM_03_02287		CNAG_06453
	Intra-centromere	1037293-1069522 (32,229)	CRAM_03_02289 (255)	Transposon	
			CRAM_03_02290 (152)	Transposon	
			CRAM_03_02291 (244)	n.a.	
			CRAM_03_02292 (230)	Transposon	
			CRAM_03_02293 (115)	n.a.	
			CRAM_03_02294 (175)	Transposon	
	Flanking (right)	1069523-1072581	CRAM_03_02295		CNAG_00393
4	Flanking (left)	227481-229236	CRAM_04_02687		CNAG_00926
	Intra-centromere	229237-277616 (48,379)	CRAM_04_02688 (67)	n.a.	
			CRAM_04_02689 (207)	Transposon	
			CRAM_04_02690 (85)	n.a.	
			CRAM_04_02691 (286)	Similar to transposase	
			CRAM_04_02692 (163)	n.a.	
			CRAM_04_02693 (116)	n.a.	
			CRAM_04_02694 (736)	Hypothetical protein	
			CRAM_04_02695 (125)	n.a.	
			CRAM_04_02696 (495)	Transposon	
			CRAM_04_02697 (747)	Transposon	
	Flanking (right)	277617-281635	CRAM_04_02698		CNAG_05242
5	Flanking (left)	330603-332956	CRAM_05_03401		CNAG_04824
	Intra-centromere	332957-371191 (38,234)	CRAM_05_03402 (122)	n.a.	
			CRAM_05_03403 (418)	Transposon	
			CRAM_05_03404 (351)	Transposon	
			CRAM_05_03405 (498)	Transposon	
			CRAM_05_03406 (135)	n.a.	
	Flanking (right)	371192-372988	CRAM_05_03407		CNAG_04853
6	Flanking (left)	787089-788876	CRAM_06_04254		CNAG_07163

Chromosome	Region	Coordinates (size; bp)	ORF (size; no. of amino acids) [1]	Description [2]	Ortholog in the H99 genome [3]
6	Intra-centromere	788876-828761 (39,884)	CRAM_06_04255 (183)	Hypothetical protein	
			CRAM_06_04256 (139)	Hypothetical protein	
			CRAM_06_04257 (328)	Transposon	
			CRAM_06_04258 (151)	Transposon	
			CRAM_06_04259 (76)	n.a.	
			CRAM_06_04260 (201)	Transposon	
			CRAM_06_04262 (68)	n.a.	
	Flanking (right)	828760-831955	CRAM_06_04263		**CNAG_02240**
7	Flanking (left)	1122672-1123409	CRAM_07_05012		CNAG_06141
	Intra-centromere	1123410-1157926 (34,516)	CRAM_07_05013 (75)	Bacterial sequence [	
	Flanking (right)	1157927-1159434	CRAM_07_05014		**CNAG_06048**
8	Flanking (left)	894669-897481	CRAM_08_05536		CNAG_05517
	Intra-centromere	897482-919853 (22,371)	CRAM_08_05537 (183)	n.a.	
			CRAM_08_05538 (110)	n.a.	
			CRAM_08_05539 (356)	Transposon	
			CRAM_08_05540 (331)	Transposon	
			CRAM_08_05541 (93)	n.a.	
			CRAM_08_05542 (243)	Transposable element	
			CRAM_08_05543 (141)	n.a.	
	Flanking (right)	919854-923991	CRAM_08_05544		**CNAG_05486**
9	Flanking (left)	821096-822472	CRAM_09_06076		CNAG_02609
	Intra-centromere	822473-859020 (36,547)	CRAM_09_06078 (128)	Mouse DNA	
			CRAM_09_06079 (205)	n.a.	
			CRAM_09_06080 (145)	n.a.	
			CRAM_09_06081 (152)	n.a.	
			CRAM_09_06082 (202)	Transposon	
	Flanking (right)	859021-860507	CRAM_09_06084		CNAG_07577
10	Flanking (left)	864022-866478	CRAM_10_06607		CNAG_00971
	Intra-centromere	866479-897364 (30,885)	CRAM_10_06608 (610)	Transposon	
			CRAM_10_06609 (418)	Transposon	
			CRAM_10_06610 (357)	Transposon	
			CRAM_10_06611 (433)	Similarity with fish DNA	
			CRAM_10_06612 (546)	Transposase	
			CRAM_10_06613 (192)	Transposon	
	Flanking (right)	897365-902058	CRAM_10_06614		**CNAG_00960**
11	Flanking (left)	610980-612932	CRAM_11_06993		**CNAG_05671**
	Intra-centromere	612543-649121 (36,578)	CRAM_11_06994 (79)	n.a.	

Chromosome	Region	Coordinates (size; bp)	ORF (size; no. of amino acids) [1]	Description [2]	Ortholog in the H99 genome [3]
			CRAM_11_06995 (297)	Transposon	
			CRAM_11_06996 (343)	Transposon	
			CRAM_11_06997 (108)	Transposon	
			CRAM_11_06998 (615)	Transposon	
			CRAM_11_06999 (97)	n.a.	
			CRAM_11_07000 (505)	Transposon	
			CRAM_11_07001 (297)	Transposon	
			CRAM_11_07002 (226)	Transposon	
			CRAM_11_07003 (188)	n.a.	
			CRAM_11_07004 (202)	n.a.	
			CRAM_11_07005 (115)	n.a.	
	Flanking (right)	649122-650987	CRAM_11_07006		**CNAG_06699**
12	Flanking (left)	467138-468905	CRAM_12_07424		CNAG_00392
	Intra-centromere	468906-513539 (44,634)	CRAM_12_07425 (136)	n.a.	
			CRAM_12_07426 (373)	Transposon	
			CRAM_12_07427 (526)	Transposon	
			CRAM_12_07428 (150)	Homology to bacterial sequence	
			CRAM_12_07429 (650)	Hypothetical protein	
			CRAM_12_07430 (357)	Hypothetical protein	
	Flanking (right)	513540-516248	CRAM_12_07432		CNAG_04869
13	Flanking (left)	601369-603529	CRAM_13_07946		CNAG_04408
	Intra-centromere	603530-642666 (39,136)	CRAM_13_07947 (153)	n.a.	
			CRAM_13_07948 (80)	Transposon	
			CRAM_13_07949 (476)	Transposon	
			CRAM_13_07950 (80)	n.a.	
	Flanking (right)	642667-654553	CRAM_13_07951		**CNAG_07769**
14	Flanking (left)	567428-570174	CRAM_14_08367		**CNAG_03258**
	Intra-centromere	570175-602631 (32,456)	CRAM_14_08369 (277)	Transposon	
			CRAM_14_08370 (624)	Transposon	
	Flanking (right)	602632-603681	CRAM_14_08371		**CNAG_03251**

[1]. Size estimation included only for those predicted ORFs that are located within the candidate centromeric regions.
[2]. n.a. indicates ORF did not return any significant hits based on BLAST of the GenBank database.
[3]. The ORFs that are in bold font are centromere-flanking in the H99 genome.
