# Supplementary material for: Fungal genome and mating system transitions facilitated by chromosomal translocations involving intercentromeric recombination
Source: PLoS Biol. 2017 Aug 11;15(8):e2002527. doi: 10.1371/journal.pbio.2002527 (PMC5568439; doi:10.1371/journal.pbio.2002527)
Supplement: S4 Table — (PDF) [file pbio.2002527.s010.pdf]

Table S4. Summary of meiotic progeny analyzed

Basidium	Cross	Germination Rate (%)	Number of progeny analyzed	Mating Type				Tetrad type (HD-PR) [1]
				A1B1	A1B2	A2B1	A2B2	
1	CBS6039 (A1B1) X CBS6273 (A2B2)	67	5	5	0	0	0	PD
2	CBS6039 (A1B1) X CBS6273 (A2B2)	57	6	0	3	3	0	NPD *
3	CBS6039 (A1B1) X CBS6273 (A2B2)	n.a.	6	4	0	0	2	PD *
4	CBS6039 (A1B1) X CBS6273 (A2B2)	81	13	0	9	4	0	NPD *
5	CBS6039 (A1B1) X CBS6273 (A2B2)	67	8	0	3	5	0	NPD
6	CBS6039 (A1B1) X CBS6273 (A2B2)	38	4	0	2	2	0	NPD or TT
7	CBS6039 (A1B1) X CBS6273 (A2B2)	89	15	6	4	3	2	TT
8	CBS6039 (A1B1) X CBS6273 (A2B2)	91	20	11	0	0	9	PD
9	CBS6039 (A1B1) X CBS6273 (A2B2)	82	16	9	0	0	7	PD
10	CBS6039 (A1B1) X CBS6273 (A2B2)	n.a.	7	0	2	0	5	TT
11	CBS6039 (A1B1) X CBS6273 (A2B2)	n.a.	9	4	1	2	2	TT
12	CBS6039 (A1B1) X CBS6273 (A2B2)	n.a.	8	5	0	2	1	TT
13	CBS6039 (A1B1) X CBS6273 (A2B2)	n.a.	8	4	0	4	0	TT
14	CBS6039 (A1B1) X CBS6273 (A2B2)	n.a.	7	2	1	2	2	TT
15	CBS6039 (A1B1) X CBS6273 (A2B2)	n.a.	7	0	0	0	7	PD or TT
16	CBS6039 (A1B1) X CBS6273 (A2B2)	n.a.	9	0	8	1	0	NPD *
17	CBS6039 (A1B1) X CBS6273 (A2B2)	n.a.	3	0	3	0	0	NPD or TT
18	CBS6039 (A1B1) X CBS6273 (A2B2)	21	3	3	0	0	0	PD or TT
19	CBS6039 (A1B1) X CBS6273 (A2B2)	38	5	0	1	4	0	NPD or TT
20	CBS6039 (A1B1) X CBS6273 (A2B2)	43	9	3	0	0	6	PD or TT
21	CBS6039 (A1B1) X CBS6273 (A2B2)	39	9	4	0	0	5	PD or TT
22	CBS6039 (A1B1) X CBS6273 (A2B2)	45	11	3	0	3	5	TT
23	CBS6039 (A1B1) X CBS6273 (A2B2)	18	4	0	0	4	0	NPD or TT
24	CBS6039 (A1B1) X CBS6273 (A2B2)	41	7	5	1	1	0	TT
25	CBS6039 (A1B1) X CBS6273 (A2B2)	n.a.	2	0	1	1	0	NPD or TT
26	CBS6039 (A1B1) X CBS6273 (A2B2)	n.a.	4	0	0	2	2	TT

Basidium	Cross	Germination Rate (%)	Number of progeny analyzed	Mating Type				Tetrad type (HD-PR) [1]
				A1B1	A1B2	A2B1	A2B2	
27	CBS6039 (A1B1) X CBS6273 (A2B2)	n.a.	3	0	0	3	0	NPD or TT
28	CBS6039 (A1B1) X CBS6273 (A2B2)	67	16	5	10	0	1	TT
29	CBS6039 (A1B1) X CBS6273 (A2B2)	89	15	4	0	0	11	PD *
30	CBS6039 (A1B1) X CBS6273 (A2B2)	78	17	4	5	4	4	TT
31	CBS6039 (A1B1) X CBS6273 (A2B2)	53	10	0	10	0	0	NPD *
32	CBS6039 (A1B1) X CBS6273 (A2B2)	53	8	4	0	0	4	PD or TT
33	CBS6039 (A1B1) X CBS6273 (A2B2)	25	3	1	0	0	2	PD or TT
34	CBS6039 (A1B1) X CBS6273 (A2B2)	21	3	3	0	0	0	PD or TT
35	CBS6039 (A1B1) X CBS6273 (A2B2)	55	12	8	3	1	0	TT
36	CBS6039 (A1B1) X CBS6273 (A2B2)	38	8	0	0	0	8	PD *
37	CBS6039 (A1B1) X CBS6273 (A2B2)	22	3	3	0	0	0	PD or TT
38	CBS6039 (A1B1) X CBS6273 (A2B2)	50	11	1	4	6	0	TT
39	CBS6039 (A1B1) X CBS6273 (A2B2)	58	15	0	1	10	4	TT
40	CBS6039 (A1B1) X CBS6273 (A2B2)	50	11	0	6	5	0	NPD *
41	CBS6039 (A1B1) X CBS6273 (A2B2)	25	6	0	5	1	0	NPD or TT
42	CBS6039 (A1B1) X CBS6273 (A2B2)	48	10	5	4	0	1	TT
43	CBS6039 (A1B1) X CBS6273 (A2B2)	42	9	3	0	0	6	PD or TT
44	CBS6039 (A1B1) X CBS6273 (A2B2)	56	12	0	0	0	12	PD *
45	CBS6039 (A1B1) X CBS6273 (A2B2)	46	12	0	3	9	0	NPD or TT
46	CBS6039 (A1B1) X CBS6273 (A2B2)	53	9	0	5	4	0	NPD or TT
47	CBS6039 (A1B1) X CBS6273 (A2B2)	52	12	0	6	6	0	NPD or TT
48	CBS6039 (A1B1) X CBS6273 (A2B2)	25	6	2	4	0	0	TT
49	CBS6039 (A1B1) X CBS6273 (A2B2)	18	3	1	0	0	2	PD or TT
50	CBS6039 (A1B1) X CBS6273 (A2B2)	53	8	0	6	2	0	NPD or TT
51	A032 (A2B2) X A067 (A1B1)	43	3	0	3	0	0	NPD *
52	A026 (A1B2) X A052 (A2B1)	21	4	2	0	2	0	TT
53	A026 (A1B2) X A058 (A2B1)	75	9	5	3	0	1	TT

Basidium	Cross	Germination Rate (%)	Number of progeny analyzed	Mating Type				Tetrad type (HD-PR) [1]
				A1B1	A1B2	A2B1	A2B2	
54	A026 (A1B2) X A058 (A2B1)	56	13	0	12	0	0	PD *
55	A026 (A1B2) X A058 (A2B1)	72	20	11	0	0	9	NPD *
56	A028 (A1B2) X A052 (A2B1)	58	7	0	7	0	0	PD *
57	A028 (A1B2) X A058 (A2B1)	76	13	3	7	2	1	TT
58	A028 (A1B2) X A058 (A2B1)	87	12	3	3	2	4	TT
59	A028 (A1B2) X A058 (A2B1)	85	9	2	0	0	7	NPD
60	A032 (A2B2) X A108 (A1B1)	91	10	8	0	2	0	TT
61	A032 (A2B2) X A108 (A1B1)	33	3	2	1	0	0	TT
62	A032 (A2B2) X A108 (A1B1)	33	4	1	3	0	0	TT
63	A790 (A1B2) X B817 (A2B1)	59	8	0	8	0	0	PD *
64	A797 (A1B2) X B821 (A2B1)	45	5	5	0	0	0	NPD *
65	A797 (A1B2) X B821 (A2B1)	20	3	0	1	2	0	PD or TT
66	A770 (A1B2) X B817 (A2B1)	67	4	0	0	4	0	PD *
67	A770 (A1B2) X B817 (A2B1)	25	2	2	0	0	0	NPD or TT
68	A770 (A1B2) X B817 (A2B1)	38	3	0	1	2	0	PD or TT
69	A770 (A1B2) X B817 (A2B1)	64	9	0	4	5	0	PD or TT
70	A790 (A1B2) X B817 (A2B1)	45	4	0	0	0	4	NPD *
71	A790 (A1B2) X B817 (A2B1)	100	10	5	4	1	0	TT
72	A790 (A1B2) X B817 (A2B1)	61	11	0	9	2	0	PD or TT
73	A790 (A1B2) X B821 (A2B1)	100	6	2	0	0	4	NPD *
74	A790 (A1B2) X B821 (A2B1)	100	12	0	3	0	9	TT
75	A790 (A1B2) X B821 (A2B1)	100	7	2	0	2	3	TT
76	A790 (A1B2) X B821 (A2B1)	100	8	3	3	2	0	TT

1. "*" indicates tetrad type determination is based on both mating type loci as well as markers located in other chromosomal regions.
