# Supplementary material for: Fungal genome and mating system transitions facilitated by chromosomal translocations involving intercentromeric recombination
Source: PLoS Biol. 2017 Aug 11;15(8):e2002527. doi: 10.1371/journal.pbio.2002527 (PMC5568439; doi:10.1371/journal.pbio.2002527)
Supplement: S5 Table — (PDF) [file pbio.2002527.s011.pdf]

Table S5. Markers and primers used in this study

Markers and Probes	Chromosome	Location (kb)	Primer (5' – 3')	
			Forward	Reverse
Genetic Markers[1]				
S2-2	2	911	JOHE37708/SS	JOHE37709/SS
Chrom10_01	10	67	JOHE41915/SS	JOHE41916/SS
Chrom10_02	10	75	JOHE41917/SS	JOHE41918/SS
Chrom10_03	10	127	JOHE41923/SS	JOHE41924/SS
Chrom10_04	10	160	JOHE37792/SS	JOHE37793/SS
Chrom10_05	10	226	JOHE41927/SS	JOHE41928/SS
Chrom10_06	10	259	JOHE37786/SS	JOHE37787/SS
Chrom10_07	10	277	JOHE38008/SS	JOHE38009/SS
Chrom10_08	10	334	JOHE38002/SS	JOHE38003/SS
Chrom10_09	10	421	JOHE41931/SS	JOHE41932/SS
Chrom10_10	10	471	JOHE37990/SS	JOHE37991/SS
Chrom10_11	10	547	JOHE37984/SS	JOHE37985/SS
Chrom10_12	10	574	JOHE27566/SS	JOHE27567/SS
Chrom10_13	10	665	JOHE24627	JOHE24630
Chrom10_14	10	678	JOHE27538/SS	JOHE27539/SS
Chrom10_15	10	698	JOHE38441/SS	JOHE38442/SS
Chrom10_16	10	704	JOHE24616	JOHE24618
Chrom10_17	10	719	JOHE27530/SS	JOHE27531/SS
Chrom10_18	10	730	JOHE27522/SS	JOHE27523/SS
Chrom10_19	10	785	JOHE38383/SS	JOHE38384/SS
Chrom10_20	10	828	JOHE41935/SS	JOHE41936/SS
Chrom10_21	10	860	JOHE38333/SS	JOHE38334/SS
Chrom10_22	10	881	JOHE38319/SS	JOHE38320/SS
Chrom10_23	10	962	JOHE37600/SS	JOHE37601/SS
Chrom10_24	10	973	JOHE37918/SS	JOHE37919/SS
Chrom10_25	10	1018	JOHE41937/SS	JOHE41938/SS
Chrom10_26	10	1062	JOHE37596/SS	JOHE37597/SS
Chrom10_27	10	1093	JOHE41903/SS	JOHE41904/SS
Chrom10_28	10	1104	JOHE41907/SS	JOHE41908/SS
Chrom10_29	10	1108	JOHE41913/SS	JOHE41914/SS
Chrom11_01	11	30	JOHE38050/SS	JOHE38051/SS
Chrom11_02	11	51	JOHE38475/SS	JOHE38476/SS
Chrom11_03	11	60	JOHE38481/SS	JOHE38482/SS
Chrom11_04	11	90	JOHE38501/SS	JOHE38502/SS
Chrom11_05	11	105	JOHE27570/SS	JOHE27571/SS
Chrom11_06	11	120	JOHE27576/SS	JOHE27577/SS
Chrom11_07	11	144	JOHE27584/SS	JOHE27585/SS
Chrom11_08	11	167	JOHE24748	JOHE24749
Chrom11_09	11	221	JOHE38068/SS	JOHE38069/SS
Chrom11_10	11	267	JOHE41939/SS	JOHE41940/SS
Chrom11_11	11	401	JOHE38092/SS	JOHE38093/SS

Markers and Probes	Chromosome	Location (kb)	Primer (5' – 3')	
			Forward	Reverse
Chrom11_12	11	408	JOHE37822/SS	JOHE37823/SS
Chrom11_13	11	450	JOHE41945/SS	JOHE41946/SS
Chrom11_14	11	469	JOHE41953/SS	JOHE41954/SS
Chrom11_15	11	510	JOHE41957/SS	JOHE41958/SS
Chrom11_16	11	607	JOHE37834/SS	JOHE37835/SS
Chrom11_17	11	656	JOHE41981/SS	JOHE41982/SS
Chrom11_18	11	713	JOHE41985/SS	JOHE41986/SS
Chrom11_19	11	742	JOHE41987/SS	JOHE41988/SS
Chrom11_20	11	787	JOHE41989/SS	JOHE41990/SS
Chrom11_21	11	826	JOHE42001/SS	JOHE42002/SS
Chrom11_22	11	847	JOHE42013/SS	JOHE42014/SS
Chrom11_23	11	902	JOHE37672/SS	JOHE37673/SS
Chrom11_24	11	1016	JOHE42027/SS	JOHE42028/SS
Chrom11_25	11	1071	JOHE42029/SS	JOHE42030/SS
Southern Probes				
Chrom01_L1	1	78	JOHE40521/SS	JOHE40522/SS
Chrom01_L2	1	1258	JOHE40523/SS	JOHE40524/SS
Chrom01_R1	1	1306	JOHE40606/SS	JOHE40607/SS
Chrom01_R2	1	2150	JOHE40601/SS	JOHE40602/SS
Chrom02_L	2	457	JOHE40611/SS	JOHE40612/SS
Chrom02_R	2	1856	JOHE40609/SS	JOHE40610/SS
Chrom03_L	3	445	JOHE40615/SS	JOHE40616/SS
Chrom03_R	3	1345	JOHE40613/SS	JOHE40614/SS
Chrom04_L	4	208	JOHE40619/SS	JOHE40620/SS
Chrom04_R	4	1248	JOHE40617/SS	JOHE40618/SS
Chrom05_L	5	216	JOHE40623/SS	JOHE40624/SS
Chrom05_R	5	1216	JOHE40621/SS	JOHE40622/SS
Chrom06_L	6	363	JOHE40625/SS	JOHE40626/SS
Chrom06_R	6	1263	JOHE40627/SS	JOHE40628/SS
Chrom07_L	7	548	JOHE40631/SS	JOHE40632/SS
Chrom07_R	7	1248	JOHE40629/SS	JOHE40630/SS
Chrom08_L	8	467	JOHE40635/SS	JOHE40636/SS
Chrom08_R	8	1167	JOHE40633/SS	JOHE40634/SS
Chrom09_L	9	562	JOHE40637/SS	JOHE40638/SS
Chrom09_R	9	962	JOHE40639/SS	JOHE40640/SS
Chrom10_L	10	262	JOHE40643/SS	JOHE40644/SS
Chrom10_R	10	962	JOHE40641/SS	JOHE40642/SS
Chrom11_L	11	345	JOHE40647/SS	JOHE40648/SS
Chrom11_R	11	716	JOHE40645/SS	JOHE40646/SS
Chrom12_L	12	40	JOHE40531/SS	JOHE40532/SS
Chrom12_R	12	984	JOHE40529/SS	JOHE40530/SS
Chrom13_L	13	240	JOHE40651/SS	JOHE40652/SS

Markers and Probes	Chromosome	Location (kb)	Primer (5' – 3')	
			Forward	Reverse
Chrom13_R	13	740	JOHE40649/SS	JOHE40650/SS
Chrom14_L	14	361	JOHE40653/SS	JOHE40654/SS
Chrom14_R	14	690	JOHE40655/SS	JOHE40656/SS
mCherry tagged Cse4 construction				
CSE4 promoter region			VYP901	VYP902
mCherry sequence			VYP903	VYP904
CSE4 ORF and terminator			VYP905	VYP906

[1]: The numbers following the underscore correspond to the marker Nos. in Figure 7.
